# Brain Gene Regulatory Networks Coordinate Nest Construction in Birds

**DOI:** 10.1093/molbev/msae125

**Published:** 2024-06-25

**Authors:** Yi-Ting Fang, Hao-Chih Kuo, Cheng-Yu Chen, Shen-Ju Chou, Chia-Wei Lu, Chih-Ming Hung

**Affiliations:** Biodiversity Research Center, Academia Sinica, Taipei, Taiwan; Department of Life Science, National Taiwan Normal University, Taipei, Taiwan; Biodiversity Research Center, Academia Sinica, Taipei, Taiwan; Biodiversity Research Center, Academia Sinica, Taipei, Taiwan; Department of Life Science, National Taiwan University, Taipei, Taiwan; Institute of Cellular and Organismic Biology, Academia Sinica, Taipei, Taiwan; Biodiversity Research Center, Academia Sinica, Taipei, Taiwan; Biodiversity Research Center, Academia Sinica, Taipei, Taiwan

**Keywords:** nest construction, transcriptome, neural plasticity, neurogenesis, pair bonding

## Abstract

Nest building is a vital behavior exhibited during breeding in birds, and is possibly induced by environmental and social cues. Although such behavioral plasticity has been hypothesized to be controlled by adult neuronal plasticity, empirical evidence, especially at the neurogenomic level, remains limited. Here, we aim to uncover the gene regulatory networks that govern avian nest construction and examine whether they are associated with circuit rewiring. We designed an experiment to dissect this complex behavior into components in response to pair bonding and nest material acquisition by manipulating the presence of mates and nest materials in 30 pairs of zebra finches. Whole-transcriptome analysis of 300 samples from five brain regions linked to avian nesting behaviors revealed nesting-associated gene expression enriched with neural rewiring functions, including neurogenesis and neuron projection. The enriched expression was observed in the motor/sensorimotor and social behavior networks of female finches, and in the dopaminergic reward system of males. Female birds exhibited predominant neurotranscriptomic changes to initiate the nesting stage, while males showed major changes after entering this stage, underscoring sex-specific roles in nesting behavior. Notably, major neurotranscriptomic changes occurred during pair bonding, with minor changes during nest material acquisition, emphasizing social interactions in nest construction. We also revealed gene expression associated with reproductive behaviors and tactile sensing for nesting behavior. This study presents novel neurogenomic evidence supporting the hypothesis of adult neural plasticity underlying avian nest-construction behavior. By uncovering the genetic toolkits involved, we offer novel insights into the evolution of animals’ innate ability to construct nests.

## Introduction

Complex adult behavioral changes are tightly linked to differential expression of genes in the brain (Zayed and Robinson 2012; York et al. 2018; Hoekstra and Robinson 2022), and adult neural plasticity can adjust behavioral phases to align with environmental and social conditions (Cardoso et al. 2015; Sinha et al. 2020). Despite this understanding, how gene regulatory networks within neural circuits orchestrate complex behavioral responses to social and environmental cues remains unclear (Sinha et al. 2020). Nest construction is a fascinating and widespread behavior among animals, particularly birds, showcasing their remarkable skill in arranging materials using their beaks (Hansell 2000). This behavior is crucial to avian evolution (Fang et al. 2018) and presumably linked to tool use (Hansell and Ruxton 2008). Birds build nests primarily during breeding, some species after finding mates, others to attract potential mates. Nest construction can involve either one or both sexes, with similar or differential tasks undertaken (Cardoso et al. 2015). The flexibility of nest-building behavior, encompassing temporal and sexual variations, makes it an ideal system to study how differential brain gene expression regulates complex behavior (Bendesky et al. 2017; Hoekstra and Robinson 2022; Hu et al. 2022). Surprisingly, the neurogenetic mechanisms governing avian nest-building behavior remain unexplored.

During breeding, zebra finches (*Taeniopygia guttata*) form monogamous pair bonds and then build nests, with males collecting nest materials and females shaping nests, often with male assistance (Zann 1996). This suggests that cues related to mates or nest materials might motivate zebra finches to construct nests. However, it is unclear whether zebra finches show different brain gene expression in response to mates and nest materials during nest construction and whether these responses are sex-specific. Examining these questions would provide insights into the neurogenetic mechanisms underlying avian nest-building behavior and other complex animal behaviors.

Flexible behavior is likely controlled by neural plasticity—changes in neuronal signaling or circuit rewiring (Zupanc and Lamprecht 2000; Cardoso et al. 2015). Studies suggest that neurotransmitters or hormones enhance synaptic transmission to induce behavioral plasticity (Taborsky and Oliveira 2012; Scharff and Adam 2013; Inada et al. 2022). Alternatively, circuit rewiring, facilitated by neurogenesis and neuron projection between brain regions, may also control behavioral plasticity (Rundstrom and Creanza 2021; Inada et al. 2022). For example, breeding songbirds increase neurogenesis and neuron projections in their song control circuit, influencing singing behavior (Troy Smith et al. 1995; Larson et al. 2013; Cohen et al. 2016; Rundstrom and Creanza 2021). Similarly, as birds also build nests during breeding, we hypothesize that nest construction is controlled by neuronal rewiring, involving generation of new neurons and formation of neuronal projections. We are particularly interested in testing whether brain gene expression changes are involved in nesting-associated neuronal plasticity.

Brain transcriptome (neurotranscriptome) profiling will be useful to understand how the crosstalk between gene expression and neural plasticity regulates nest construction (Sinha et al. 2020). Traditional analyses of neuron expression provide limited insights into the genetic mechanism that regulates behavior. Numerous brain gene expression studies use whole brains and therefore lack region-specific neurotranscriptomic insights tied to behavior. Hence, we aim to profile transcriptomes from specific brain regions associated with avian nest-building behavior to reveal the genetic regulatory mechanism of this behavior. The brain regions we focused on include the anterior motor pathway (AMP), the social behavior network (SBN), and the dopaminergic reward system (dopaminergic neuron population, DNP), as previous studies suggested that their neuronal activities are related to regulating nest-building behavior in zebra finches (Hall et al. 2014; Edwards et al. 2020). In addition, considering the importance of the beak in nest construction (Sheard et al. 2023), we also hypothesize that tactile sense in the beak is critical to avian nest building. Thus, investigating gene expression in the pons and medulla (PM; i.e. brainstem), which convey tactile-related sensorimotor signals from and to the beak (Gutiérrez-Ibáñez et al. 2009; Wild and Krützfeldt 2012; Schneider et al. 2014), could help assess the role of tactile sense in nest construction.

To study the neurotranscriptomic mechanism of neural plasticity driving nest-building behavior, we analyze transcriptomes in the four key brain regions related to nest-building in male and female zebra finches, alongside corresponding behavioral data. Given the complexity of nest-building behavior, we designed experiments to dissect neurotranscriptomic profiles into mate- or nest material–induced components. This distinction is achieved by comparing nesting finches that stayed with their mates and had access to nest materials with finches that were separated from either mates or nest materials. Our study provides the first view on how sex-specific gene regulatory networks interact with social and environmental cues to rewire neural circuits, governing bird nest construction. Additionally, we identify pivotal genes in regulating this behavior, improving our understanding of what genes drive animals to build nests.

## Results

### Nest Construction Actions Induced by Mates and Nest Materials

**Fig. 1.**
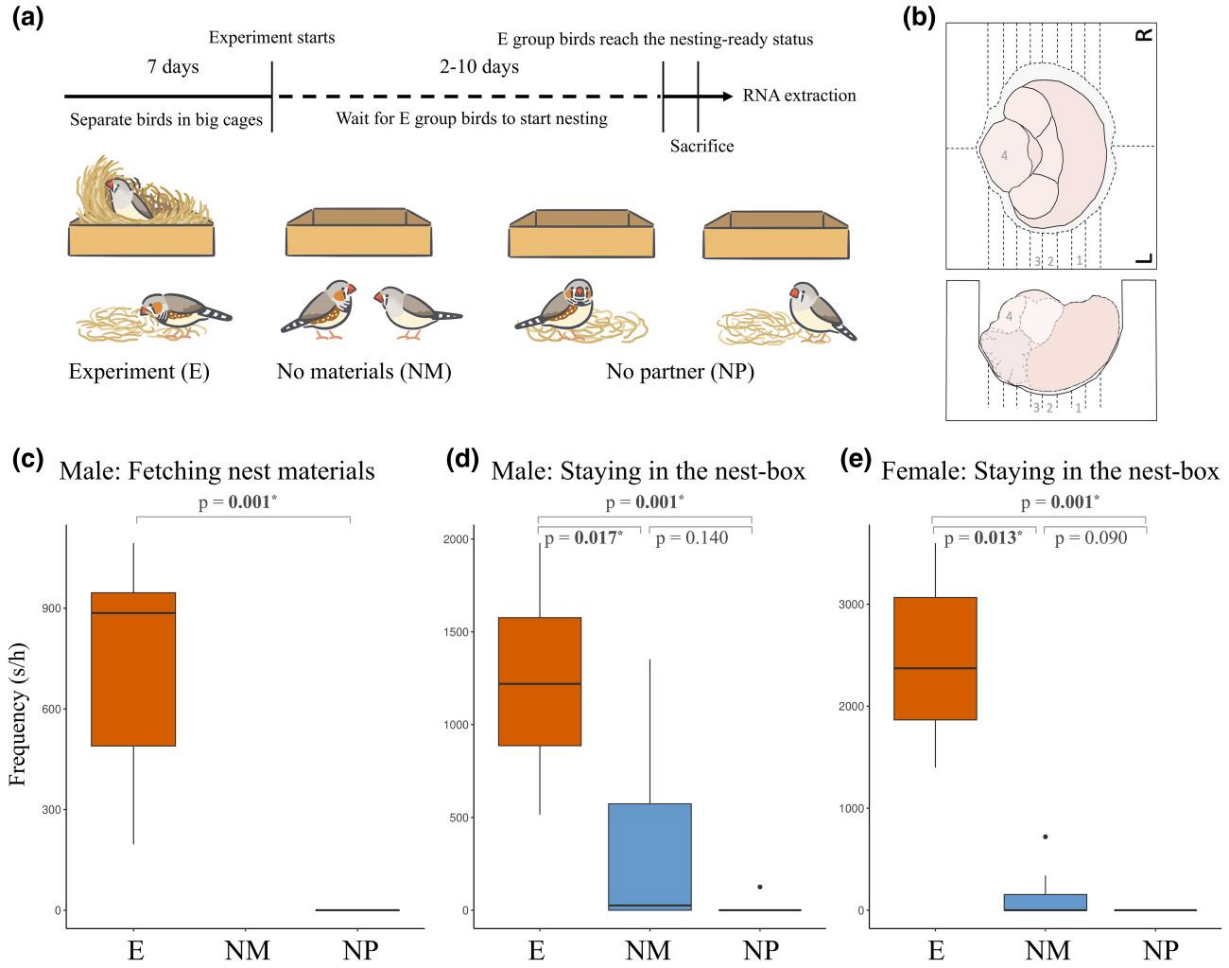
Nesting behavior experimental design and behavioral analyses. a) Test trial setting and workflow for the experiment. In the experiment (E) group, finches stayed together with their mates and had access to nest materials. In the NM group, finches stayed together with their mates but lacked nest materials. In the NP group, finches had access to nest materials but were separated from their mates. b) The anatomical landmarks of a zebra finch brain placed upside down in a brain matrix from the top-view (upper panel) and lateral-view perspectives (lower pane). The numbers indicate the sections where 4 specific brain regions collected for RNA-seq analysis are located: (1) AMP, (2) SBN, (3) DNP, and (4) PM. c–e) Comparison of the frequencies of 3 nesting-related actions among the E, NM, and NP groups: c) fetching nest materials in male birds, d) staying in the nest-box in male birds, and e) staying in the nest-box in female birds. The Benjamini–Hochberg adjusted *P*-values of Mann–Whitney U tests are shown and significant *P*-values are in boldface (*P < 0.05). Central lines, box limits, whiskers, and points in box-plots indicate medians, upper and lower quartiles, 1.5 × interquartile ranges, and outliers, respectively. Illustrations of the nesting behavior experiment by Hsiang-Ching Chen.

We conducted a nesting experiment involving 30 pairs of zebra finches divided into 3 treatments: experimental (E), no-material (NM), and no-partner (NP) groups (Fig. 1a). In the E group, 10 pairs stayed together with their mates and had access to nest materials. In the NM group, 10 pairs stayed together with their mates but lacked nest materials, while in the NP group, 10 pairs had access to nest materials but were separated from their mates. We found no nest constructed by any birds in the NM or NP groups. To assess behavioral differences, we recorded and quantified 11 actions, comprising nesting-related actions and others, in the 60 studied birds for 80 min (Methods, supplementary table S1, Supplementary Material online). Subsequently, we collected their brain tissues for RNA sequencing (Fig. 1b). We found that male birds in the E group exhibited significantly higher frequencies of fetching nest materials and staying in the nest-box (site) than birds from the NM or NP groups (Mann–Whitney *U* test, Benjamini–Hochberg adjusted *P* < 0.02; Figs. 1c and d). Similarly, female birds in the E group showed significantly higher frequencies of staying in the nest-box (Benjamini–Hochberg adjusted *P* < 0.01; Fig. 1e). However, we found no significant differences in nest-box occupancy between birds from the NM and NP groups, regardless of sex. None of the remaining 9 actions showed significant frequency differences among the 3 treatment groups, except for a lower frequency of drinking action in the females of the E group than those of the NP group (Benjamini–Hochberg adjusted *P* = 0.015; supplementary table S2, Supplementary Material online). Notably, actions related to courtship (allopreen, beak fencing, and upright fluffed singing) and copulation were not significantly different between the birds from the E and NM groups. Among the birds of the E group, the usage of nest materials was marginally correlated with the frequency of male fetching action, but not with that of males or females staying in the nest-box (supplementary fig. S1, table S1; Supplementary Results, Supplementary Material online).

### Neurotranscriptomic Clusters-matched Anatomical Regions

**Fig. 2.**
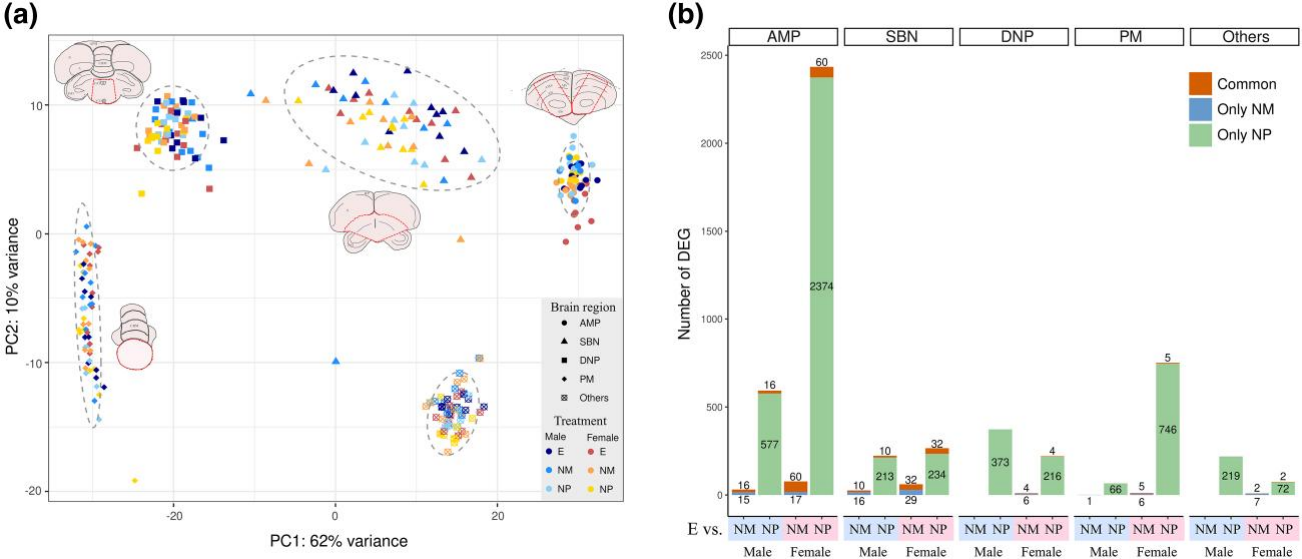
Brain transcriptomic clusters and differential expressed genes (DEGs). a) PCA clusters of transcriptomes from 5 brain regions. Different colors indicate three treatments (E, NM, and NP) and two genders, and different shapes indicate the 5 brain regions (AMP, SBN, DNP, PM, and Others). Brain section images are positioned near the transcriptomic clusters of their corresponding brain regions (see supplementary fig. S2, Supplementary Material online, for the detailed descriptions of the images). b) The numbers of DEGs resulted from the comparisons of E versus NM and E versus NP for each gender and brain region. DEGs that are exclusive to either the E versus NM or E versus NP comparison, as well as those that are common to both comparisons, are represented separately.

To examine the transcriptomic mechanism underlying nest construction, we conducted RNA-seq analysis on brain RNA samples collected from the 60 studied birds. We collected 5 brain regions, including AMP, SBN, DNP, PM, and Others (containing the remaining parts of brain tissues), resulting in a total of 300 samples (Methods, Supplementary Results, Supplementary Material online, supplementary fig. S2, table S3, Supplementary Material online), all of which exhibited similar distributions of expression levels for the total genes analyzed (supplementary fig. S3, Supplementary Material online). The brain’s regional transcriptomic differences were revealed by the principal component analysis (PCA) of whole-transcriptome data with PC1 explaining 62% of the variance and PC2 explaining 10% (22,150 genes; Fig. 2a). However, samples of the 3 treatment groups were mixed in each PCA cluster, indicating minor effect of nest construction on the whole transcriptomes of the brain regions.

### Nesting-associated Neurotranscriptomes Exhibited a Female-Skewed Gene Count

To identify genes associated with the transition between nesting and nonnesting stages, we compared the nesting group with each of the 2 nonnesting groups (E-NM and ENP) using differential gene expression tests. In our experimental setup, each trial comprised one E, one NM, and one NP pair (Methods), which was factored into the modeling of differential gene expression tests. The approach allowed us to control for conditional differences across trials, thereby enhancing the identification of differentially expression genes (DEGs) between treatment groups. We detected a total of 1,169 and 3,083 DEGs (Benjamini–Hochberg adjusted *P* < 0.05) in male and female birds, respectively, with an equitable distribution between up-regulated and down-regulated genes (supplementary table S4, Supplementary Material online). The number of DEGs in females was around 2.5 times higher than in males for both E-NM and E-NP comparisons (supplementary Results, Supplementary Material online). In the E-NP comparison, we found the female-skewed patterns in all brain regions and treatment comparisons except for the brain regions titled DNP and Others (Fig. 2b, Supplementary Results, Supplementary Material online). The results suggest that females exhibit stronger neurotranscriptomic changes in most of the nest-associated brain regions compared with males during the transitions from nonnesting to nesting stages.

### Mate Influence Outweighed Nest Materials in Nesting-associated Neurotranscriptomes

Among the 1,169 DEGs in male birds, 1,123 genes were exclusively identified between the birds in the E and NP groups (E-NP-specific DEGs), 31 genes between birds in the E and NM groups (E-NM-specific DEGs), and 26 genes in the birds of both the NP and NM groups compared with those in the E group (common DEGs; Fig. 2b, supplementary table S4, Supplementary Material online). In female birds, 2,978 genes were E-NP-specific DEGs, 54 genes were E-NM-specific DEGs, and 91 genes were common DEGs (Fig. 2b, supplementary table S4, Supplementary Material online). The more the E-NP-specific DEGs than the E-NM-specific DEGs, the stronger the influence of mates compared with nest materials on brain gene expression associated with nest construction. This implies that (re-)establishing pair bonds may play a larger role than obtaining nest materials in preparing zebra finches neurologically for nest building.

### The Anterior Motor Pathway Showed Dominant Nesting-associated Neurotranscriptomic Response

Interestingly, while the 5 brain regions showed similar gene expression levels (supplementary fig. S3, Supplementary Material online), the AMP exhibited up to 10 times more DEGs, including E-NP-specific, E-NM-specific, and common DEGs, than other brain regions (SBN, DNP, PM, or Others) for both male and female birds (Fig. 2b, Supplementary Results, supplementary table S4, Supplementary Material online). The results suggest the dominant role of AMP in controlling transition between nesting and nonnesting life history stages in zebra finches. In addition, the analysis of the whole brain (“Others”) yielded fewer DEGs than individual brain regions, emphasizing the significance of examining specific brain regions for accurate neurotranscriptomic analysis.

### Neurotranscriptomes Revealed the Role of Circuit Rewiring in Bird Nest Construction

**Fig. 3.**
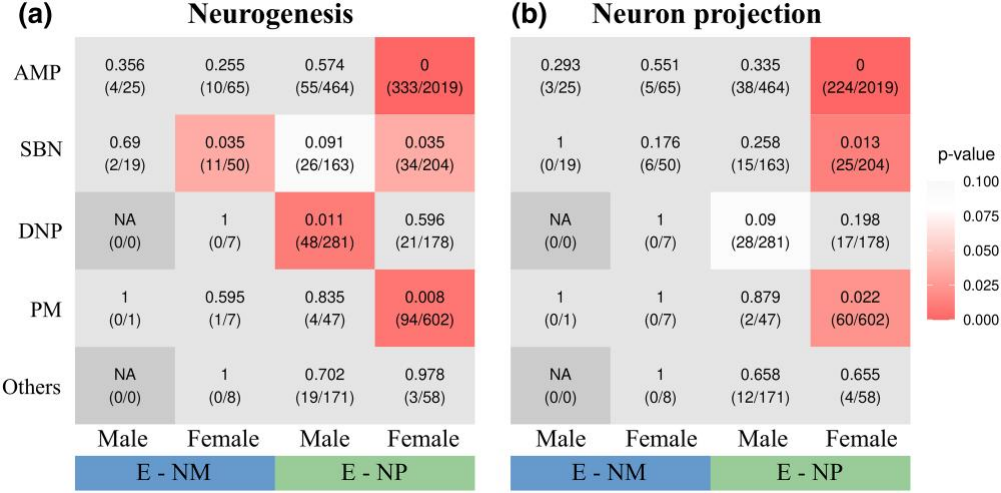
Over-representation of differentially expressed genes (DEGs) in neural rewiring functions. Over-representation in a) neurogenesis and b) neuron projection functions for DEGs from 5 brain regions and based on comparisons between E versus NM and E versus NP for each gender. The numbers within each cell indicate the *P*-values of over-representation analysis (upper values) and the number of DEGs belonging to the respective functional category/the total number of DEGs in each brain region and treatment (values within brackets).

We then examined DEGs in each brain region to investigate the molecular mechanism underlying neural circuit dynamics in nest construction. Neural circuits rewiring, a crucial role in behavioral control, can occur through neuronal addition and/or modification of neuronal projection (Zupanc and Lamprecht 2000; Cardoso et al. 2015). To assess the impact of neurogenesis and neuron projection on circuit alteration during nesting, we devised an approach to detect over-representation of DEGs in 2 Gene Ontology (GO) categories: “neurogenesis” and “neuron projection” (72 GO terms in total; Methods; supplementary table S5, Supplementary Material online). In the E-NM comparison for males, neither neurogenesis nor neuron projection functions were over-represented in DEGs across the 5 brain regions; however, in females, we found over-representation of neurogenesis function in the SBN region (*P* = 0.035; Fig. 3, supplementary table S6, Supplementary Material online). By contrast, in the E-NP comparison among males, the neurogenesis function was over-represented in the DEGs of DNP (*P* = 0.011, Fig. 3A). For females in the E-NP comparison, both neurogenesis and neuron projection functions were over-represented in the DEGs of AMP (*P* < 0.001 in both functions), SBN (*P* = 0.035 and 0.013, respectively), and PM (*P* = 0.008 and 0.022, respectively; Fig. 3, supplementary table S6, Supplementary Material online).

These results suggest that the addition of neurons and neuron projections contributes to the rewiring of nesting-associated circuits, a pattern steadily detectable in females, when birds are forming pair bonds rather than obtaining nest materials. Additionally, varying levels of support were gained across different brain regions. In AMP and PM, which regulate motor actions and tactile-based sensorimotor functions (Gutiérrez-Ibáñez et al. 2009; Wild and Krützfeldt 2012; Schneider et al. 2014), respectively, only female E-NP DEGs showed significant enrichment in neurogenesis and neuron projection. Thus, stronger evidence was obtained for the influence of mate-associated cues than of nest materials in reconstructing motor/sensorimotor circuitries for nest building in female finches. We acknowledge that the lack of support for over-representation of neurogenesis/neuron projection in males may stem from lower statistical power caused by the smaller number of DEGs. Nevertheless, the higher the number of neurogenesis/neuron projection DEGs in females compared with males (Fig. 3), the more robust the gene expression profile associated with these functions in females.

The SBN influences various social behaviors in birds, including courtship singing (Heimovics and Riters 2006), copulation (Balthazart and Surlemont 1990), incubation (Youngren et al. 1989), parental care (Fazekas et al. 2020), and nest building (Hall et al. 2014, 2015; Kingsbury et al. 2015). DNP, closely connected to SBN as part of a social decision-making network (O'Connell and Hofmann 2011b), motivates and reinforces these behaviors (Charlier et al. 2005; Heimovics and Riters 2005; Banerjee et al. 2013; Hall et al. 2014, 2015; Kingsbury et al. 2015). Studies suggest that neurons in SBN and DNP influence these social behaviors via signaling molecules such as vasopressin, oxytocin, or dopamine (O'Connell and Hofmann 2011a; Hall et al. 2015; Kingsbury et al. 2015). Our results suggest that circuit rewiring in SBN and DNP also regulates nest construction in zebra finches. However, only female SBN and male DNP showed DEG enrichment in neurogenesis and/or neuron projection, suggesting different rewiring patterns in the social decision-making networks between female and male finches during the onset of the nesting stage. Interestingly, both female E-NM and E-NP DEGs were enriched with neurogenesis in SBN, suggesting that both mate- and nest material–associated cues affect circuit rewiring in female SBN. It highlights neuronal addition to SBN as a core neurological mechanism underlying female nesting behaviors, including pair bond establishment and nest material manipulation.

We further used the over-representation analysis to show no significant over-representation of in situ neurogenesis function in DEGs across any examined brain regions in male or female birds (*P* > 0.05, supplementary fig. S4, table S6; Supplementary Results, Supplementary Material online). Additionally, the DEGs of the regions collectively called Others were not over-represented with neurogenesis, neuron projection, or in situ neurogenesis in any treatment comparison for either sex.

### Neurotranscriptomes Revealed Physiological and Behavioral Traits Associated With the Target Brain Regions and Nesting Stage

We performed enrichment analysis for DEGs using general GO terms, not limited to those associated with neural-circuit-related functions, to investigate broad biological functions related to the nesting stage. As expected from the DEG counts, more enriched terms were identified in the E-NP comparison than E-NM (supplementary table S7, Supplementary Material online). Furthermore, several enriched terms were related to the functions of target brain regions, validating the accuracy of our RNA sampling approach. For example, female E-NP DEGs were enriched with terms such as “locomotory behavior”, “associative learning”, and “learning or memory” in AMP, which is known to govern body movement and learning behavior, such as song learning (Feenders et al. 2008; Andalman and Fee 2009; Zhao et al. 2023). In PM (brainstem), female E-NP DEGs were enriched with the terms “detection of mechanical stimulus involved in sensory perception of pain” and “positive regulation of heart contraction”, consistent with the roles of the brainstem in pain processing (Napadow et al. 2019) and heart rate control (Dergacheva et al. 2014; Aiba et al. 2016), respectively. The female E-NP DEGs of PM were also enriched with “response to corticosterone”, supporting the integration of corticosterone signals in controlling autonomic function by the brainstem (Ragozzino et al. 2020). Additionally, a behavioral term “copulation” was enriched in female E-NP DEGs in PM, known to regulate copulation solicitation display in female songbirds (Perkes et al. 2019).

In SBN, hormone-related terms “positive regulation of cortisol secretion” and “positive regulation of glucocorticoid secretion” were enriched in female and male E-NP DEGs, respectively, consistent with the role of the hypothalamus (parts of SBN) in regulating corticoid secretion in the adrenal cortex (Tortorella et al. 2007; Tadross et al. 2010). The female E-NP DEGs of SBN were also enriched with GO terms “ovulation cycle process”, “estrous cycle”, and “cellular response to follicle-stimulating hormone stimulus”, all of which are regulated through a cascade of reactions initiated by the gonadotropin-releasing hormone generated in the hypothalamus (Herbison 2020; Gutierrez-Castellanos et al. 2022). The result is consistent with the reported association between nest-building behavior and follicle-stimulating hormone in the ring dove (*Streptopelia risoria*; Cheng and Balthazart 1982).

In DNP, DEGs were enriched with several GO terms linked to the functions of dopaminergic neurons in the ventral tegmental area (VTA) of this brain region. For example, 2 enriched terms in male E-NP DEGs, “fear response” and “regulation of corticosterone secretion”, related to the fear learning mechanism (Tang et al. 2000) and corticotropin-releasing hormone actions (Zalachoras et al. 2022), respectively, both regulated by VTA dopaminergic neurons. In addition, female E-NP DEGs were enriched in “maternal aggressive behavior” and males in “locomotory exploration behavior”, corresponding to the roles of VTA dopaminergic neurons in mediating aggressive behavior (Mahadevia et al. 2021) and exploration behavior toward novel stimuli (Bariselli et al. 2018; Shan et al. 2023), respectively. The aggressive and exploration behaviors could be associated with gender-specific nesting behaviors (see Discussion).

### Gene Co-expression Modules Linked to Nesting Onset Also Displayed Female-skewed and Mate-induced Neural Rewiring Enrichment

We applied weighted gene co-expression network analysis (WGCNA), which estimated expression correlations among individual genes, to cluster 18,056 genes from all 300 samples into 40 co-expression gene modules (supplementary table S8, Supplementary Material online). We then applied the over-representation test to identify modules related to neuron projection and neurogenesis (Methods). Among the 40 modules, 2 were enriched in both neuron projection–related and neurogenesis-related genes, while 2 modules were only enriched in neurogenesis-related genes (supplementary fig. S5, table S9, Supplementary Material online).

We identified WGCNA modules that differed between nesting (E) and nonnesting (either NM or NP) birds in the expression correlation level among constituting genes, indicating altered expression connectivity strength. Such modules, identified using modular differential connectivity (MDC; Zhang et al. 2013) estimates, were considered functionally relevant to nesting stage shifts (Bagot et al. 2016) and labeled as “MDC modules” (supplementary table S10, Supplementary Material online). In the examined brain regions (excluding Others), females and males had similar numbers of MDC modules (module × brain region = 59 for females and 58 for males). Additionally, the E-NP comparison had a similar number of MDC modules compared with the E-NM comparison (58 for E-NP and 59 for E-NM; supplementary fig. S5, Supplementary Results, Supplementary Material online).

Remarkably, female birds had nearly 2 times more MDC modules associated with neurogenesis and/or neuron projection in specific brain regions (excluding Others; module × brain region = 8 for neurogenesis and 3 for neuron projection) compared with male birds (4 for neurogenesis and 2 for neuron projection; supplementary fig. S5, Supplementary Material online). Similarly, the E-NP comparison showed nearly 2 times more MDC modules associated with neurogenesis and/or neuron projection in those brain regions (8 for neurogenesis and 3 for neuron projection) compared with the E-NM one (4 for neurogenesis and 2 for neuron projection). Especially, female MDC modules were enriched with neurogenesis/neuron projection in more brain regions (i.e. SBN, DNP, and PM) than male ones (i.e. SBN and DNP; supplementary fig. S5, Supplementary Material online). These MDC module findings align with the DEG results (Fig. 3), indicating stronger neurotranscriptomic responses associated with circuit rewiring in females and to mates when finches shift to the nesting stage.

We also applied separated WGCNA to male and female datasets and identified MDC modules from the gender-specific WGCNA modules (Supplementary Results, Supplementary Material online). We found similar patterns of the MDC modules between the analyses conducted with combined and separated genders (supplementary fig. S5 and S6, Supplementary Results, Supplementary Material online). In particular, the female-skewed and mate-induced neural rewiring enrichment persisted regardless of whether male and female data were combined or separated for WGCNA module construction.

### Gene Co-expression Modules Correlated With Nesting Action Frequencies Differed From Modules Linked to Nesting Onset

**Fig. 4.**
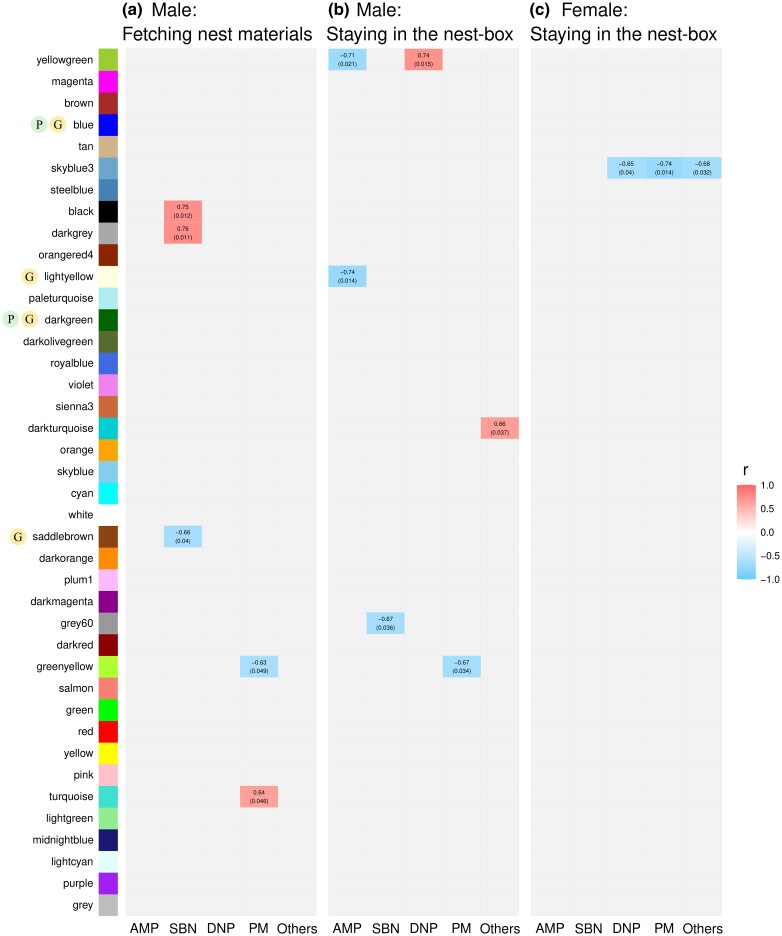
Co-expression gene modules correlated to nesting behavior frequencies (NBF modules). The colored cells indicate co-expression modules with significant correlations with the frequency of a) fetching nest materials in males, b) staying in the nest-box in males, or c) staying in the nest-box in females in each brain region. The red color of the boxes indicates significantly positive correlation modules, and the blue color of the boxes indicates significantly negative correlation modules. Numbers within each cell indicate the correlation coefficient and the *P*-values (within brackets) of correlation analysis. The modules over-represented with neuron projection and neurogenesis functions are marked with Ⓟ and Ⓖ, respectively.

Genomic mechanisms governing nesting onset and maintaining individual nest-building actions may differ. To examine this, we identified WGCNA modules correlated with nest-building action frequencies. We estimated module eigengenes (MEs), which characterized module-specific expression profiles, using the first principal components of individual modules (supplementary table S11, Supplementary Material online). We then correlated MEs with the frequencies of nest-material fetching and nest-box staying in male E birds, as well as nest-box staying in female E birds, across brain regions. We denoted modules that showed significant correlations with the nest-building behavior frequencies as “NBF modules” and considered them as the regulators of these behaviors. We detected NBF modules for male staying-in-nest action in more brain regions (AMP, SBN, DNP, and PM, excluding Others) than for female staying-in-nest action (DNP and PM) or male fetching action (SBN and PM; Fig. 4, Supplementary Results, Supplementary Material online). Similar patterns were observed when we estimated NBF modules from gender-specific WGNCA modules (supplementary fig. S7, Supplementary Results, Supplementary Material online). Hereafter, we only reported the results derived from gender-combined analysis.

In SBN, one NBF (saddlebrown) module for male fetching action was associated with neurogenesis (Fig. 4a), and another module (black) was enriched for neural signaling functions (supplementary table S12, Supplementary Material online). Similarly, one NBF (lightyellow) module for male staying-in-nest action in AMP was also associated with neurogenesis (Fig. 4b), and enriched for neural signaling functions, including dopaminergic synaptic transmission (supplementary table S12, Supplementary Material online). By contrast, the only NBF module for female staying-in-nest action was enriched for immune-related, but not neuron-related, functions (supplementary table S12, Supplementary Material online). The results suggest that during the nesting period, male fetching and staying-in-nest behaviors are regulated by neural signaling and neurogenesis, whereas female staying-in-nest behavior is less likely to be influenced. The results contrast with the female-skewed pattern of neural plasticity in DEGs and MDC modules associated with the initiation of nesting.

NBF modules showed minimal overlap with MDC modules except for one module (supplementary fig. S8, Supplementary Material online). This finding strengthens the evidence that gene regulatory networks governing the transition between nesting and nonnesting stages largely differ from those maintaining nesting action frequencies. Previous studies also revealed distinct neural activity patterns for nesting onset compared with nesting action variations (Hall et al. 2014, 2015).

### Social Hormone DEGs Were Female-skewed and Strongly Mate-induced, While Social Hormone NBF Modules Were Male-skewed

We found several female E-NP-specific DEGs associated with the social hormone (i.e. oxytocin, vasopressin, and dopamine) signaling, known to influence nesting behaviors in birds and mice (Klatt and Goodson 2013; Hall et al. 2015; Bendesky et al. 2017). For example, *OXT* in DNP and *AVP* in AMP encode oxytocin and vasopressin, respectively (supplementary table S4, Supplementary Material online). *AVPR1A* and *AVPR2*, encoding vasopressin receptors, were identified in PM and AMP, SBN and DNP, respectively; *DRD1*, *DRD2,* and *DRD5* in AMP encode dopamine receptors (supplementary table S4, Supplementary Results, Supplementary Material online).

One NBF (turquoise) module for male fetching behavior identified in PM contained *AVPR1A*, *DRD3*, and *DBH*, which regulates the dopamine-to-norepinephrine conversion (Kim et al. 2002; supplementary table S8, Supplementary Material online). In AMP, one NBF (light yellow) module for male staying-in-nest behavior contained tyrosine hydroxylase (*TH*) (supplementary table S8, Supplementary Material online) involved in dopamine synthesis (Daubner et al. 2011), and enriched in dopaminergic synaptic transmission function (supplementary table S12, Supplementary Material online).

The above results suggest the involvement of oxytocinergic, vasopressinergic, and/or dopaminergic systems in (i) preparing female zebra finches for initiating the nesting stage, induced by mates, and (ii) maintaining male nest construction actions in the nesting stage.

### DEGs, MDC and NBF Modules Associated With Tactile Sensorimotor Signaling in PM and Beyond

We identified E-NP-specific DEGs related to the tactile sense, such as *DRGX* and *TLX3,* in the female PM region (supplementary table S4, Supplementary Material online). *DRGX*, regulated by *TLX3*, is a transcription factor patterning whisker-specific neuronal structure—barrelettes—in the principal trigeminal nucleus (PrV) of mice (Erzurumlu et al. 2010). We also identified tactile-related, E-NP-specific DEGs in brain regions other than PM that were part of NBF or MDC modules identified in PM. For example, in male AMP, we identified *PIEZO2*, which encodes a mechanosensitive ion channel and is critical for processing tactile information from duck beaks (Schneider et al. 2017; see other examples in Supplementary Results, Supplementary Material online). Among these DEGs, *PIEZO2*, and *ASIC2* were part of an NBF (turquoise) module for fetching action in male PM (Fig. 4, supplementary table S8, Supplementary Material online). *ASIC2*, *NTRK2*, and *PCDH15* were included in one (blue) module and *LHFPL5* in another (brown) module, both of which were MDC modules for the E-NP comparison in female PM (supplementary fig. S5, table S8, Supplementary Material online).

### Hub Genes of NBF and MDC Modules

To identify the key drivers of the NBF and MDC modules, we analyzed their hub genes (Methods). We particularly focused on 1 fetching-associated NBF (turquoise) module in male PM because it included 2 tactile-related DEGs—*PIEZO2* and *ASIC2*—and 3 social behavior-related DEGs—*AVPR1A*, *DRD3*, and *DBH* (Fig. 4, supplementary table S8, Supplementary Material online). *LPIN1*, a transcriptional coactivator impacting motor neuron development (Péterfy et al. 2010; Lu et al. 2021), emerged as a hub gene in this module (supplementary table S13, Supplementary Material online). Another hub gene, *SLC6A11*, encodes the GABA-transporter 3 (Kersanté et al. 2013), which regulates GABA, a major neurotransmitter modifying neural firing patterns, network activity, and synaptic plasticity (Foster and Kemp 2006), and influences the grooming behavior of rodents (Zink et al. 2009; Yu et al. 2018). Our results suggest that *SLC6A11* and *LPIN1*, two neuron-related genes, may also influence the fetching behavior of male nesting birds through interactions with the tactile sense and social decision-making network. In addition, we observed a significant association between the hub genes of MDC or NBF modules and gene transcription functionality (Supplementary Results; supplementary table S13 and S14, Supplementary Material online).

## Discussion

The ability of animals to adapt their reproductive behaviors to fit environmental changes (e.g. building nests when conditions are right) has long fascinated scientists. However, the neurotranscriptomic mechanism underlying this behavioral plasticity remains unclear. Studies have attributed adult behavioral plasticity to 2 types of neural plasticity: structural reorganization to form new neural circuits and biochemical switching of neuromodulatory molecules within existing circuits (Zupanc and Lamprecht 2000; Cardoso et al. 2015). While the switching of neural signals has been extensively studied, recent studies have uncovered evidence of neurogenesis and neural projection that rewires circuits, leading to behavioral changes (Gheusi et al. 2009; Larson et al. 2013; Cohen et al. 2016; Düring et al. 2020). However, few studies have examined the transcriptomic mechanism responsible for neural structural and neurohormonal changes in response to external factors that drives behavioral plasticity. Here, we utilized whole-transcriptome analyses across multiple brain regions combined with behavioral experiments to identify neuron-related genes and co-expression modules associated with nest-building behavior in zebra finches. We discovered that (i) finches displayed stronger neurotranscriptomic changes for circuit rewiring in response to cues from mates than nest materials. Furthermore, (ii) female birds formed more extensive circuit rewiring signals in motor/sensorimotor and social behavior networks during transition to the nesting stage while males showed stronger signals when maintaining nest construction frequency. Our findings provide the first evidence of brain-wide transcriptomic responses associated with avian nest building, reflecting various forms of neural plasticity.

It has been proposed that adult behavioral changes involve alternations in neural circuits and require the generation of new neurons to strengthen circuit wiring (Zupanc and Lamprecht 2000; Cardoso et al. 2015). Our results suggest that circuit rewiring regulates the nesting behavior of zebra finches, supported by transcriptomic enrichment of neurogenesis and neural projection functions in motor/sensorimotor and social decision-making circuitries. However, we found no evidence of in situ neurogenesis in the examined brain regions, implying that new neurons may migrate from other regions. Similarly, during song behavior, white-crowned sparrows (*Zonotrichia leucophrys*) exhibit new neuron migration to the high vocal center, from which the neurons project to the robust nucleus of the arcopallium (Larson et al. 2013; Cohen et al. 2016). Adult black-capped chickadees (*Poecile atricapillus*) add new neurons to the hippocampus during winter and spring when they need to remember and retrieve stored food (Sherry and Hoshooley 2010). Our findings suggest that the neuronal number in the motor/sensorimotor and social behavior networks of zebra finches might also increase during the nesting stage.

Adult neurogenesis during the nesting period suggests that nesting behavior relies on a complex neural structure involving new neurons and projections, especially in female finches. These findings imply that maintaining the necessary neuron connections for nesting behavior during nonbreeding periods is costly. Brain tissue needs about 10 times more energy than muscle tissue (Balasubramanian 2021), highlighting the energetic cost of nesting behavior at both the organismal and neural levels. Thus, neural plasticity likely serves as a mechanism for birds to conserve energy during transitions between different behavioral stages throughout the year.

Our results suggest more extensive neurotranscriptomic changes in response to mate-related cues compared with nest materials. This could be attributed to 2 factors. One is that social interactions require complex behavioral responses and thereby elicit more neurotranscriptomic changes (Cardoso et al. 2015). If so, this pattern should be limited to the social decision-making network (SBN and DNP); however, we observed this pattern across all examined brain regions. Alternatively, neural circuits involved in nesting behaviors largely overlap with those governing pair bonding. Zebra finches form monogamous pair bonds, through which males and females engage in collaborative nest building (Zann 1996). The nest construction process includes a nest ceremony characterized by courtship-like movements, which strengthen the pair bond (Zann 1996). This explains the consistent patterns across brain regions associated with social behaviors (SBN and DNP), sequential motor actions (AMP), and sensorimotor reactions (PM). Additionally, we found no significant differences in courtship and copulation behaviors between the birds of the E and NM groups, both with access to mates. This, along with the greater impact of mates compared with nest materials on neurotranscriptomic changes related to circuit rewiring, suggests that mate-induced gene expression may largely prepare birds for subsequent nest construction, pending the availability of nest materials. Once the necessary nest-related circuits are formed and the birds are physically prepared for nesting, minor neurotranscriptomic changes induced by nest materials may suffice to initiate nest building. Our results suggest that the minor changes in circuit rewiring induced by nest materials likely occur in female SBN (Fig. 3a). This efficient strategy allows paired birds to start constructing nests as soon as they find suitable materials. Furthermore, the neurotranscriptomic changes likely reflect the temporal order of reproductive behaviors—with pair bonding preceding nest construction—characterizing the life history of zebra finches and many other songbirds.

In addition to enriched expression of circuit rewiring in response to pair bonding, we identified E-NP-specific DEGs associated with social hormones like *OXT* and *AVP*, as well as their receptors (*AVPR2, AVPR1A, DRD1*, *DRD2*, and *DRD5*). These neuromodulators have been found to regulate nesting behaviors in zebra finches and mice (Hansell and Ruxton 2008; Klatt and Goodson 2013; Hall et al. 2015) and pair bonding in voles (Lim et al. 2004). Our results suggest that these neuromodulators may contribute to avian nesting behavior by influencing social interactions rather than directly initiating nest-building because they are DEGs responding to mate-related cues, not nest materials. This suggests that vasopressin, oxytocin, and dopamine may control both pair bonding and nest building in zebra finches.

We found that male and female zebra finches exhibit distinct neurotranscriptomic changes associated with nest construction. Reproduction strategies often differ between genders, resulting in varied responses to social and environmental cues that regulate their reproduction behaviors (Ball and Ketterson 2008). Studies have reported sex-specific neural and transcriptomic activations involved in social (Shepard et al. 2009; Dumais and Veenema 2016) and nesting behaviors (Klatt and Goodson 2013; Hall et al. 2014; Kingsbury et al. 2015; Bendesky et al. 2017). We found that female zebra finches exhibit more DEGs and MDC modules enriched in circuit rewiring functions and/or containing social hormone-related genes in most brain regions in response largely to mate-related cues. By contrast, males exhibit more NBF modules with similar functional enrichment and genes. In addition, only DNP shows a higher likelihood of DEG enrichment with circuit rewiring in male zebra finches, suggesting their reliance on dopaminergic reward system rewiring for nesting onset.

Different nesting-associated tasks between male and female zebra finches may cause their distinct neurotranscriptomic changes. This is further emphasized by the enrichment of GO terms related to behavioral or physiological traits in gender-specific E-NP DEGs. Although male zebra finches are more active in courtship and nest construction, females have the final say in pair formation and exert effort to maintain or re-establish the pair bond, closely tied to the nesting stage (Zann 1996). Interestingly, female E-NP DEGs in PM were enriched with the GO term “copulation”, which may relate to the copulation solicitation display of female birds (Perkes et al. 2019), thus underscoring the decisive role of female birds in pair formation. Additionally, female birds undergo stronger physiological changes for reproduction, including oviposition, which is supported by the enrichments of GO terms “ovulation cycle process” and “estrous cycle” in the female E-NP DEGs of SBN. One GO term “maternal aggressive behavior” was enriched in the female E-NP DEGs of DNP, implying that the neurotranscriptomic change elevates aggression in nesting females for protecting their forthcoming offspring or defending nest sites. Conversely, the male E-NP DEGs of DNP were enriched with “locomotory exploration behavior”, which includes exploration toward novel stimuli (Bariselli et al. 2018; Shan et al. 2023) and may drive male birds to search for nest materials. In summary, our results highlight the distinct neural and physiological mechanisms underlying nesting behaviors in female and male zebra finches, with female investing more energy in pair bonding, physiologically preparing for the nesting stage, and defending nest sites, while males contribute more during nest construction by collecting nest materials.

We also analyze the transcriptomic profiling of PM to explore the association between the tactile-based sensorimotor circuitry and nest-building behavior. Interestingly, 2 female DEGs in PM are related to patterning of tactile-related neuron modules in the PrV of mice. Additionally, 2 MDC modules in female PM and 1 NBF module in male PM contain tactile-related genes. Surprisingly, several tactile-related DEGs are expressed in brain regions outside of PM, especially in AMP. Notably, male DEGs in AMP are enriched with a GO term “detection of mechanical stimulus involved sensory perception” (supplementary table S7, Supplementary Material online). Among the DEGs associated with this GO term, *PIEZO2* encodes pressure-activated ion channels sensitive to subtle physical force and is expressed in the prefrontal cortex and other brain regions in mice (Wang and Hamill 2021); in addition, *PIEZO2* is involved in processing tactile sense from duck beaks (Schneider et al. 2017), implying its role in controlling delicate actions such as fetching nest materials. Thus, we reason that tactile-related, mechanosensitive ion channels expressed in brain regions beyond PM may also modulate tactile-related neuronal signals during nest construction.

## Conclusion

Hansell and Ruxton (2008) argue that nest construction and tool use are strongly associated, both being construction behaviors. Zebra finches’ nest-building behavior involves anterior striatum (ASt) (in AMP), which activates the basal ganglia for tool use in Japanese macaques (*Macaca fuscata*; Obayashi et al. 2001; Hall et al. 2014). Tool use in birds may involve the mesopallium, nidopallium, and striatopallidal complex (Mehlhorn et al. 2010), the anterior part of which is AMP. AMP governs sequential motor actions and has evolved to regulate avian song behavior (Feenders et al. 2008). Given the observed prominent transcriptomic changes in this region, which are also enriched with GO terms of “locomotory behavior”, “associative learning”, and “learning or memory”, we suggest a comparable regulatory mechanism for nest building during evolution. Additionally, the caudolateral nidopallium (NCL) integrates sensorimotor signals including touch sense for tool use in birds (Striedter 2013). Although we did not examine NCL in this study, our results of dominant gene expression patterns in AMP and involvement of the tactile-based sensorimotor circuitry suggest that avian nest building shares neural similarity with tool use (AMP and PM). However, nest construction involves additional circuitries (SBN and DNP), highlighting a degree of divergence in the underlying mechanisms between these behaviors. The differing mechanisms likely correspond to the distinct impacts of pair bonding on nest construction and tool use. Our study also underscores the importance of investigating specific brain regions, as opposed to the whole brain, to unveil the neurotranscriptomic mechanism of behavior.

## Materials and Methods

### Animals

The subjects for the study included 30 male and 30 female zebra finches. The care and use of birds were approved by the Institutional Animal Care and Use Committee of Academia Sinica (Prove ID: 17-05-1096).

### Experimental Animals and Laboratory Settings

We conducted a nesting experiment using 30 pairs of zebra finches, all aged more than 166 days post hatching (dph; supplementary table S15, Supplementary Material online) and with a history of successful nesting behaviors. Since zebra finches typically reach sexual maturity at 3 months of age and the median age of first breeding is 92 to 95 dph (Zann 1996), our studied birds were all considered adults. Furthermore, prior to the nesting experiment, all 30 pairs of zebra finches had built nests in an open nest-box (15 cm × 15 cm × 3.5 cm) located within a breeding cage (45 cm × 40 cm × 40 cm). Before starting the formal experiments, we temporarily separated the studied birds from their mating partners and housed them in large cages (1 m × 1 m × 1 m) with other birds of the same sexes and with each cage accommodating a maximum of 10 finches. The large-cage phase lasted for at least 1 week. After this large-cage phase, we reintroduced the birds to individual breeding cages, each equipped with an open nest-box, to conduct the nesting experiments.

We maintained specific environmental conditions for both the bird rooms housing the breeding cages and the large cages. In the room with breeding cages, we set the light cycle to 14 light hours (5:00 to 19:00) followed by 10 dark hours (19:00 to 5:00), kept the humidity within a range of 40% to 70%, and maintained the temperature between 23 and 25 °C throughout the years. The other bird room housing the large cages had the same conditions as the breeding room, except for one adjusted light cycle of 13 light hours (5:30 to 18:30) and 11 dark hours (18:30 to 5:30). All birds had *ad labium* access to water and food.

### Nesting Behavior Experiment and Brain Tissue Collection

We randomly divided the 30 pairs of birds to three test groups, each comprising 10 pairs (Fig. 1a): (i) In the experiment (E) group, we placed one pair of birds in a single breeding cage with provided nest materials. (ii) The NM group consisted of pairs housed in a breeding cage without nest materials. (iii) In the NP group, we separated 1 pair of birds into 2 breeding cages, both equipped with nest materials. For the E and NP groups, we replenished the cages with 10 g of coconut fibers as nest materials daily. The daily usage of nest materials was weighted between 10:00 AM and 10:10 AM throughout the experiment. Any unused coconut fibers were replaced with fresh 10 g of coconut fibers each day.

We conducted a series of 10 experimental trials, each comprising 3 groups—E, NM, and NP. In each experimental trial, we defined the E group as performing nesting behavior based on 2 criteria: (i) Nesting-ready status—if birds either used a total of > 10 g of nest materials for 3 successive days or used > 3 g on each of these 3 successive days, we considered them to have reached the “nesting-ready status”. (ii) Nesting behavior confirmation—once birds attained the “nesting-ready status”, we confirmed nesting behavior if, on any given morning during the status, male birds collected nest materials into the nest-box and female birds stayed in the nest-box between 10:10 AM and 11:30 AM. Upon meeting both of these conditions for the E group birds, we collected brain samples from all birds in the E, NM, and NP groups at 12:00 PM (noon) or 12:30 PM on the same day. It required well trained operators to precisely collect brain samples for RNA extraction, and we had 3 such operators. Thus, we could collect samples from only 3 out of the 6 birds simultaneously. The first 3 birds were sampled at 12:00 PM and the second 3 at 12:30 PM. We carefully designed the sampling schedule for the 10 experimental trials to ensure even distribution of the sampling orders across the E, NM, and NP groups, and between sexes.

During the sampling procedure, we extracted a whole brain from the cranial cavity and dissected it using a bird brain matrix (Huang et al. 2019). We collected 5 brain regions (Fig. 1b, supplementary fig. S2, Supplementary Material online), 3 of which have been found related to nesting behavior (Hall et al. 2014). These 3 regions included (i) AMP, which contains anterior ventral mesopallium, anterior nidopallium, and ASt, (ii) SBN, which encompasses the bed nucleus of the stria terminalis, medial preoptic area (POM), anterior hypothalamus, and ventromedial hypothalamus, and (iii) DNP, which comprises the VTA and central gray of the mesencephalon. In addition, we hypothesized that the tactile sense was critical to fetching nest materials and constructing nests. Thus, we extended our investigation to encompass (4) PM, colloquially referred to as the brainstem. Within this region, the principal trigeminal nucleus (PrV) processes tactile information from the beak, palate, and tongue (Gutiérrez-Ibáñez et al. 2009; Schneider et al. 2014), and relays it to the nucleus basorostralis (Bas) in the rostrobasal part of the avian forebrain (Wild 2015). In addition, the motor output from Bas may reach the parvocellular reticular formation (RPcvm) within the brainstem, an area housing premotor neurons responsible for regulating beak movements in songbirds (Wild and Krützfeldt 2012). Finally, we collected (5) the remaining brain tissues, denoted simply as “Others”. The brain tissues were preserved in RNA later at −80 °C until RNA extraction.

#### Behavior Quantification and Statistic

We recorded the nest building actions of zebra finches using AW-720CIP (Jinwei Electronic) cameras in the morning, precisely from 10:10′00 AM to 11:29′59 AM, before collecting their brain tissue samples. A total of 80 min were recorded for each of the 30 pairs, except for one pair in which the recording from 11:17′00 AM to 11:20′59 AM was lost. We analyzed the behavior videos based on one-second windows. We measured 2 actions related to nest building, including fetching nest materials (followed by placing them into the nest-box), and staying in the nest-box. Although we could not distinguish whether they shaped the nest or simply possessed the nest site during the “stay in the nest-box” period, we considered both as part of nesting behavior. We also measured 9 other actions, including upright fluffed singing, copulating, beak wiping, allopreening, autopreening, tail-quivering, beak fencing, feeding, and water demanding (drinking). We then estimated the frequency of each action, representing the number of seconds each action occurred per hour (supplementary table S1, Supplementary Material online).

To assess whether the behaviors were different among groups subjected to different experimental treatments, we used Mann–Whitney *U* tests to compare the frequency of each action between pairs of treatments (i.e. E and NM, E and NP, NM and NP), separately for male and female birds (supplementary table S2, Supplementary Material online). We applied the Benjamini–Hochberg correction to the *P*-values to take into account for multiple comparisons involving both the numbers of nest building actions and treatment comparisons.

We also conducted the Pearson correlation analysis to investigate the potential correlation between the weight of nest materials used during nest construction and the frequencies of fetching action in male birds or staying in the nest-box in each gender. The analysis focused only on birds from the E group, which were engaged in nest building. We assessed the usage of nest materials based on 2 definitions. First, we examined nest material usage specifically on the morning of brain sample collection (sacrifice day). Secondly, we considered nest materials used during the entire experimental trials, beginning with the presence of nest materials in the nest-box. One E pair, named R5 (supplementary table S1, Supplementary Material online), was excluded from the analysis due to their depletion of the initial 10 g of nest materials, after which they received an additional 10 g on the morning of the sacrifice day.

#### cDNA Library Preparation and Sequencing

We extracted total RNA from the abovementioned 5 brain regions using the RNeasy Fibrous Tissue Mini Kit (Catalog No. 74704; Qiagen) following manufacturer's instructions. RNA-seq libraries of 300 samples (60 birds × 5 brain regions) were prepared following the Illumina TruSeq Stranded mRNA library preparation workflow (Genomics, Taiwan). We performed paired-end sequencing (2 × 150 bp) on the Illumina NovaSeq 6000 platform (Genomics, Taiwan) to the RNA-seq libraries.

### Transcriptome Assembly and Annotation

We trimmed off adapters and low-quality sites from raw sequencing reads using Trimmomatic version 0.38 with the following parameters: HEADCROP:10 LEADING:22 TRAILING:22 SLIDINGWINDOW:6:22 MINLEN:36. We mapped the trimmed reads to the zebra finch reference genome (Ensemble: bTaeGut1_v1.p) using Hisat2 version 2.1.0 (Kim et al. 2015) with –rna-strandness set to RF. To estimate the expression levels of genes, we first estimated the average per-base sequencing coverage of each transcript (for a total of 38,869 transcripts) using StringTie version 2.1.4 (Pertea et al. 2015) with the parameter –rf. We then converted the coverage to read counts (= coverage ×transcript length/read length) for each transcript and estimated read counts for each gene (a total of 22,150 genes) using a python script provided by the StringTie website. The read count estimate was required for the negative binomial generalized linear models used for differentially expressed gene analysis (see the following for details).

For functional annotation and downstream analyses, we sought to determine the HUGO Gene Nomenclature Committee (HGNC) symbols of all protein-coding genes in the zebra finch genome (bTaeGut1_v1.p). To achieve this, we mapped transcript protein sequences of zebra finches, downloaded from the Ensembl genome browser 103, to vertebrate proteins in the eggNOG database using eggNOG-mapper V2 (Huerta-Cepas et al. 2017; Huerta-Cepas et al. 2019) with the default settings. We then combined the eggNOG annotated result with the Ensembl 103 BioMart data, prioritizing Ensembl data, and we successfully mapped HGNC symbols to 14,889 out of 16,619 protein-coding genes.

### Clustering Analysis of Gene Expression Pattern

To assess transcriptomic responses across different brain regions and genders in the three treatment groups (E, NM, and NP), we conducted the PCA on the expression data encompassing all 22,150 genes in the zebra finch genome. Prior to PCA, we applied a variance-stabilizing transformation to the gene counts. We conducted the PCA analyses using the Deseq2 package (Love et al. 2014) in R.

#### DEGs Between Nesting and Nonnesting Birds

We identified DEGs between the nesting (E) and NM groups and between the E and NP groups, with gender and brain regions analyzed separately. To enhance the robustness of our analysis and reduce noise introduced by genes with low expression levels, we implemented two steps: (i) We excluded genes with an average expression level of lower than one read count per gene per sample. That is, we only considered genes with a minimum total expression of 30 read counts across all samples in each brain region and gender. (ii) We also removed genes that showed zero read count in more than 10 out of 30 samples for each brain region and gender. Subsequently, we normalized read count data based on the median-of-ratios method (Anders and Huber 2010) for conducting the generalized linear model (GLM)-based differential gene expression analysis using Deseq2. To control for conditional differences among experimental trials, we incorporated “trial” as an additional factor in GLMs. For significant assessment, we set Benjamini–Hochberg adjusted *P*-values < 0.05 as the thresholds for defining DEGs.

While there existed a few seeming outliers in the PCA clusters corresponding to brain regions (Fig. 2a), we did not remove these samples from DEG analysis for several reasons. First, arbitrarily removing outliers could introduce unaware biases into our analysis. Secondly, our experiment design involved pairing one E group with an NM group and an NP group (i.e. the experimental trials) and differential expression analysis was conducted with this factor included in GLMs. Removing outliers would force us to abandon such paired-sample information, which typically leads to lower power in detecting DEGs. Thirdly, we performed additional analysis by removing 2 seeming outliers, 1 from SBN, and 1 from PM (supplementary fig. S9a, Supplementary Material online), and conducted tests based on GLMs without including the trial factor. We found that the distribution pattern of DEGs (supplementary fig. S9b, Supplementary Material online) remained similar to the one without removing the seeming outliers (Fig. 2b). Especially, AMP exhibited the largest numbers of DEGs and females had more DEGs than males in AMP, SBN, and PM. However, more comparisons resulted in zero DEGs, which made downstream analyses more difficult to conduct. Consequently, we opted to conduct DEG analysis on the original dataset.

### Over-representation Analyses of Neural Function Genes

To test whether genes expressed in the examined brain regions during the nesting process were associated with the alteration of neural circuits, we developed an over-representation analysis. First, we categorized functions for all zebra finch genes using ConsensusPathDB (CPDB; http://cpdb.mol-gen.mpg.de/; Kamburov et al. 2009) to obtain sets of GO terms at levels 4 and 5. Focusing on broad processes of neural circuit rewiring, we identified GO terms associated with neural-circuit-related functions in the Biological Process category. We classified the GO terms into 3 custom categories—neuron projection (including 36 GO terms and 900 HGNC gene symbols), in situ neurogenesis (including 7 GO terms and 91 HGNC gene symbols), and other neurogenesis actions (including 29 GO terms and 1,440 HGNC gene symbols; supplementary table S5, Supplementary Material online). The “neuron projection” category encompassed GO terms associated with the development or extension of axons or dendrites, and axon projection guidance. The “in situ neurogenesis” category included GO terms related to the generation or division of neuronal stem cells or neuroblasts. The “other neurogenesis actions” process category comprised GO terms associated with neuron migration, differentiation, or development, including the broader concept of neurogenesis.

We then tested whether the DEGs identified earlier were over-represented in functions related to (i) neural projection and/or (ii) neurogenesis (combining categories “in situ neurogenesis” and “other neurogenesis actions” with 36 GO terms and 1,453 HGNC gene symbols). We conducted the one-tailed Fisher exact test to examine whether the numbers of DEGs in these categories outnumbered those not included while considering the background genes as the genes expressed in each respective brain region (the same set of genes used in the DEG identification analysis). For the brain regions that had DEGs over-represented in functions of neurogenesis, we further tested whether the neurons were generated in situ by applying the same over-representation test to the “in situ neurogenesis” category. We considered a result as significant if the *P*-value was < 0.05.

It should be noted that the hierarchical structure of the GO system rendered the GO terms within the “neuron projection” category also falling under the higher-level GO term—Neurogenesis (GO:0022008)—which belonged to the “neurogenesis” category. Consequently, genes assigned to the “neuron projection” category based on their GO terms would be also counted in the “neurogenesis category”, but not the other way around.

### GO Term Enrichment Test for DEGs

To comprehensively examine the biological functions influenced by gene expression in different brain regions under our experimental conditions, we performed general GO term enrichment analysis for the DEGs. This analysis was not limited to neural-circuit-related functions. We annotated GO terms to all genes using eggNOG-mapper V2, and performed the enrichment analysis using the topGO package in R. We employed Fisher's exact tests for the enrichment analysis and utilized the “elim” algorithm as an alternative to conventional multiple-comparison correction methods (Alexa et al. 2006; Alexa and Rahnenfuhrer 2010). We focused solely on Biological Process terms and considered GO terms with a *P*-value < 0.01 as significantly enriched.

#### Weighted Gene Co-expression Network Analysis

We applied the WGCNA to identify gene co-expression modules using the *WGCNA* package (Langfelder and Horvath 2008) in R. A WGCNA module referred to a cluster of genes exhibiting high expression correlations, suggesting interactive relationships among these constituting genes. Gene expression levels were estimated and presented in the unit of transcripts per kilobase million (TPM) provided by StringTie, and then we log_2_ transformed the expression values. Genes with no expression (TPM = 0) in more than 20 out of 60 samples in the same brain regions were removed from the analysis, leaving a total of 18,056 genes for the WGCNA analysis. We constructed gene expression networks using the *TOMsimilarityFromExpr* function with a soft thresholding power of 6, opting for the “signed hybrid” network type and “bicor” correlation type. The WGCNA module construction encompassed all 300 samples, representing 5 brain regions from 60 birds. In addition to the comprehensive WGCNA across all 300 samples, we also constructed gender-specific WGCNA modules using datasets specific to males and females, each comprising 150 samples, to examine the impact of gender separation on the analysis.

We then examined whether the WGCNA modules were associated with the function of neuron projection and/or neurogenesis by using the over-representation analysis. Specially, we conducted the one-tailed Fisher exact test to identify modules that were over-represented by genes belonging to the category of “neuron projection” or “neurogenesis”, consistent with the approach used in the DEG over-representation analysis. We corrected the *P*-values for multiple comparisons involving the number of modules using Benjamini–Hochberg adjustment.

Furthermore, for a comprehensive understanding of the biological functions associated with the WGCNA modules, we conducted GO term enrichment analysis for each module. The GO term enrichment approach was the same as that used for DEGs. Briefly, we annotated GO terms to all constituting genes of a module using eggNOG-mapper V2, and performed Fisher's exact tests for the enrichment analysis utilizing the “elim” algorism of the topGO package. We set a *P*-value < 0.01 as the threshold to determine significance and only considered Biological Process terms.

### WGCNA Modules With MDC Between Nesting and Nonnesting Birds

It has been assumed that gene modules showing alteration in expression connectivity (i.e. the correlation level of expression among constituting genes) between different states of a behavior are functionally relevant to that behavior (Bagot et al. 2016). In other words, the genes within these modules reshape their intra-modular relationship during the exhibition of the behavior. Thus, we reasoned that WGCNA modules that showed varying strength of expression connectivity between nesting and nonnesting conditions may be critical to the physiological or neural underpinnings of nesting condition setup. To identify these critical modules, we estimated MDC (modified from Zhang et al. 2013) between the E and NP groups and between the E and NM groups for each WGCNA module in each brain region of the male or female birds. First, we estimated intra-modular connectivity (*kwithin*: the sum of the Pearson correlation coefficients between the expression levels of a particular gene and those of all other genes within the same module) for every gene in a given module (referred to as X module) separately for different brain regions, sexes, and treatment groups. Secondly, we calculated MDC of X module using the following formula:


$$\mathrm{MDC}=\frac{\begin{array}{c} \mathrm{sum}(kwithin\;\mathrm{of}\;\mathrm{every}\;\mathrm{genes}\;\mathrm{in}\;X\;\\ module\;in\;the\;E\;group) \end{array}}{\begin{array}{c} \mathrm{sum}(kwithin\;\mathrm{of}\;\mathrm{every}\;\mathrm{genes}\;\mathrm{in}\;X\;\\ module\;in\;the\;NM\;or\;NP\;group) \end{array}},$$

MDC > 1 indicated an increase in expression connectivity among genes in the module during nesting, whereas MDC < 1 indicated a reduction in the expression connectivity during nesting. Third, we assessed statistical significance of MDC by using a permutation test to distinguish between two scenarios—gain of connectivity (MDC > 1) and loss of connectivity (MDC < 1). Given *M* permutations, the *P*-value of MDC > 1 or < 1 was computed as follows:


$$\mathrm{gain}:P(\mathrm{MDC}>1)=1-\left(\frac{1}{M}\sum_{\;p=1}^{M}\mathrm{MDC}>\;\mathrm{MD}C^{p}\right),$$

$$\mathrm{loss}:P(\mathrm{MDC}<1)=1-\left(\frac{1}{M}\sum_{\;p=1}^{M}\mathrm{MDC}<\;\mathrm{MD}C^{p}\right).$$

where $\mathrm{MD}C^{p}$ was MDC derived from a permutated dataset. We permutated the *kwithin* values between the E and NM/NP groups 10,000 times (*M* = 10,000), with the resultant *P*-value subject to Benjamini–Hochberg adjustment based on the number of modules examined. We defined a module as showing significant gain of connectivity if its Benjamini–Hochberg adjusted *P* < 0.05 and MDC > 2, and a module as showing significant loss of connectivity if its Benjamini–Hochberg adjusted *P* < 0.05 and MDC < 0.5, which represents at least twice the difference between two treatment groups. We denoted these modules as MDC modules, which play a crucial role in initiating the nesting stage.

### WGCNA Modules Associated With the Frequencies of Nesting Behaviors

To identify modules that may regulate nesting behavior intensity, we first calculated MEs, which represent module-specific expression profiles derived from the first principal component of the gene expression matrix of each module (Langfelder and Horvath 2008). These MEs were estimated separately for each combination of treatments, genders, and brain regions, resulting in 10 samples for each distinct combination. Subsequently, we conducted the Pearson correlation analysis in the E group to examine the relationships between MEs and the frequencies of nesting-related actions. These actions were fetching nest materials and staying in the nest-box for the male birds, and staying in the nest-box for the female birds. We assumed modules that showed significant correlation with these action frequencies as potential regulators of “nesting behavior frequencies” and denoted them as NBF module.

### Hub Genes of WGCNA Modules

To pinpoint the key genes governing the nesting-related modules, particularly (i) those showing significant MDC between nesting and nonnesting states (MDC modules) or (ii) those associated with NBF modules, we identified hub genes within these modules. We calculated module membership (MM; Langfelder and Horvath 2008) for all genes within the modules for the E group in each brain region for both male and female birds. We also calculated gene significance (GS; Langfelder and Horvath 2008) for all genes within the modules for (i) the E-NM and E-NP comparisons when examining MDC modules or (ii) only the E group when examining NBF modules. MM quantified how close a gene is to the module it belongs to, measured by the Pearson correlation coefficient between its gene expression level and the module eigengene. GS gauged the association of a gene with a biological trait. We quantified GS of a gene against a nesting behavioral trait with a Pearson correlation coefficient between its gene expression level and (i) nesting versus nonnesting states or (ii) the frequency of fetching nest materials or staying in the nest-box. We identified hub genes based on criteria of |MM| > 0.8 (Chen et al. 2018; Laine et al. 2019) and |GS| > 0.51 for MDC modules (corresponding to the top 1% of |GS| values in MDC modules) or |MM| > 0.8 and |GS| > 0.77 for NBF modules (corresponding to the top 1% of |GS| values in NBF modules).

## Supplementary Material

msae125_Supplementary_Data

## Data Availability

The RNA-seq reads used in this study will be deposited in the NCBI Sequence Read Archive under the access number PRJNA1124911. Other datasets used in this study are available in supplementary tables, Supplementary Material online. The codes used in this study are available in the Figshare repository (https://figshare.com/s/b353a6796d0587d71da2).
