# Supplementary material for: Brain Gene Regulatory Networks Coordinate Nest Construction in Birds
Source: Mol Biol Evol. 2024 Jun 25;41(7):msae125. doi: 10.1093/molbev/msae125 (PMC11223658; doi:10.1093/molbev/msae125)
Supplement: msae125_Supplementary_Data [file msae125_supplementary_data.zip › Supplementary Figures_MBE_Final.pdf]

Brain gene regulatory networks coordinate nest construction in birds

Yi-Ting Fang[1,2], Hao-Chih Kuo[1]†, Cheng-Yu Chen[1,3]†, Shen-Ju Chou[4]†, Chia-Wei Lu[1] and Chih-Ming Hung[1]*

[1] Biodiversity Research Center, Academia Sinica, Taipei, Taiwan

[2] Department of Life Science, National Taiwan Normal University, Taipei, Taiwan

[3] Department of Life Science, National Taiwan University, Taipei, Taiwan

[4] Institute of Cellular and Organismic Biology, Academia Sinica, Taipei, Taiwan

* Corresponding author: Chih-Ming Hung

Email: cmhung@gate.sinica.edu.tw

† These authors contributed equally

Supplementary Figures

Fig. S1-S9

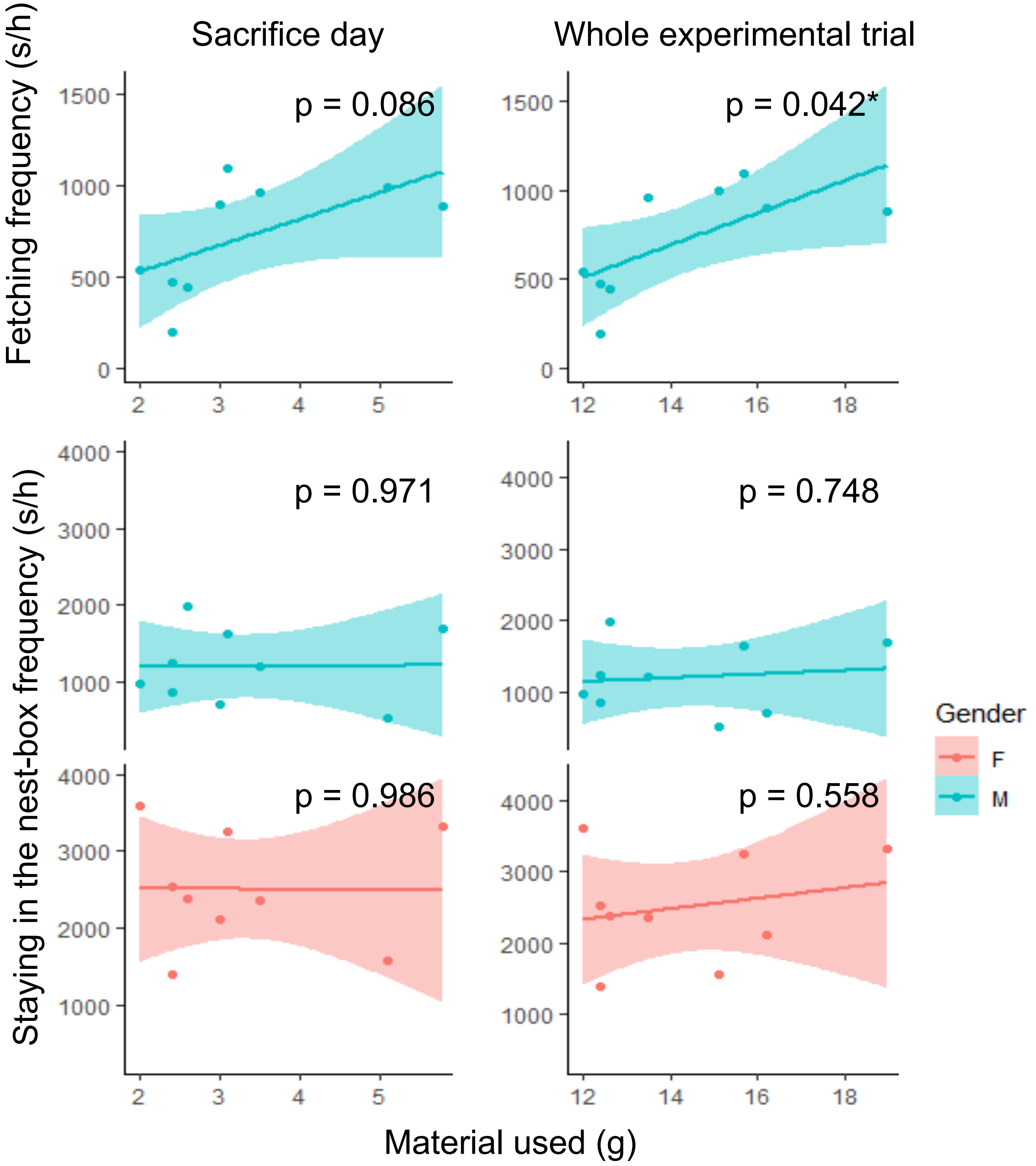

Figure S1. The relationship between the usage of nest materials and the frequencies of nest construction actions. The Pearson correlation analysis is used to investigate the relationship between the weight of nest materials used during nest construction and the frequencies of fetching action in male birds or staying in the nest-box in each gender from the E group. The usage of nest material is defined in the ways: (1) the nest materials used specifically on the morning of brain sample collection (sacrifice day) and (2) the materials used during the entire experimental trials, beginning with the presence of nest materials in the nest-box (whole experiment trial). * indicates $p < 0.05$.

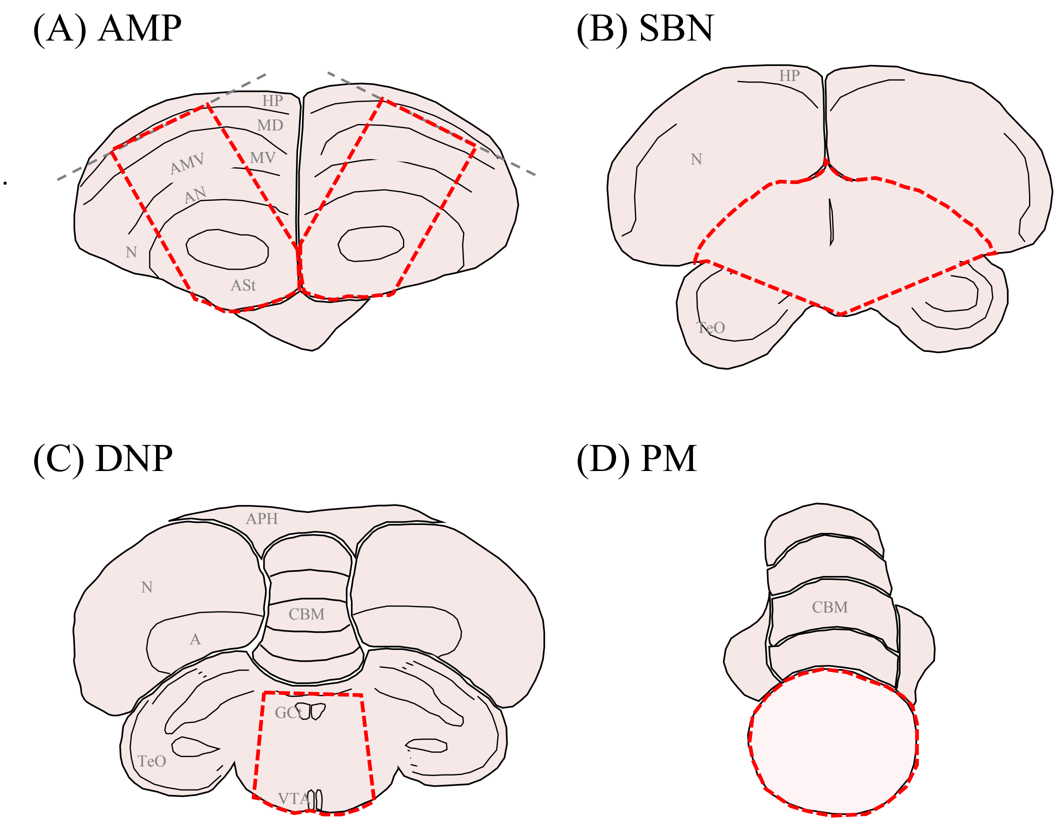

Figure S2. Brain regions sampled for RNA-seq analysis to represent the (a) anterior motor pathway (AMP), (b) social behavior network (SBN), (c) dopaminergic neuron population (DNP) and (d) pons and medulla (PM) in the zebra finch. Areas within red dash line indicated the region we sampled. Abbreviations: A = arcopallium; AMV = anterior ventral mesopallium; AN = anterior nidopallium; APH = parahippocampal areas; ASt = anterior striatum; CBM = cerebellum; GCt = central gray of the mesencephalon; HP = hippocampus; MD = dorsal mesopallium; MV = ventral mesopallium; N = Nidopallium; TeO = tectum opticum.

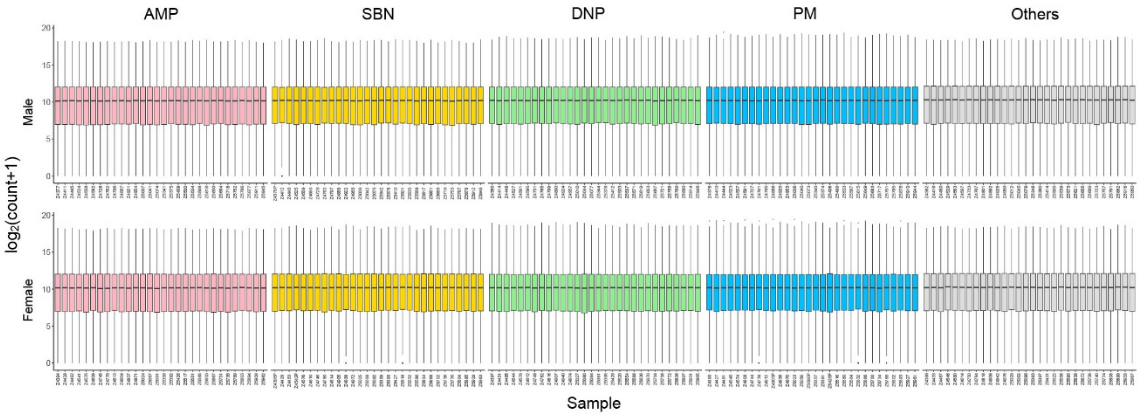

Figure S3. Distribution of gene expression levels across 300 samples from five brain regions and both sexes. The analyzed genes are the same as those used as the input data for DEG analysis in this study. The gene expression levels are represented by read counts in every sample after normalization with respect to sample library sizes in a $\log_2$ scale.

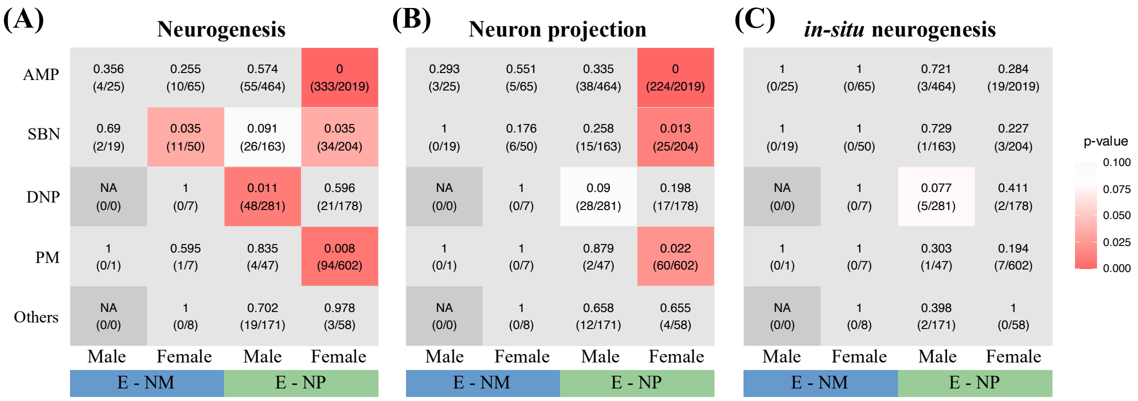

Figure S4. Over-representation of differentially expressed genes (DEGs) in neural rewiring functions. Over-representation in (A) neurogenesis, (B) neuron projection and (C) *in situ* neurogenesis functions for DEGs from five brain regions and based on comparisons between E vs. NM and E vs. NP for each gender. Numbers within each cell indicate the p-values of over-presentation analysis (upper values) and the number of DEGs belonging to the respective functional category / the total number of DEGs in each brain region and treatment (values within brackets).

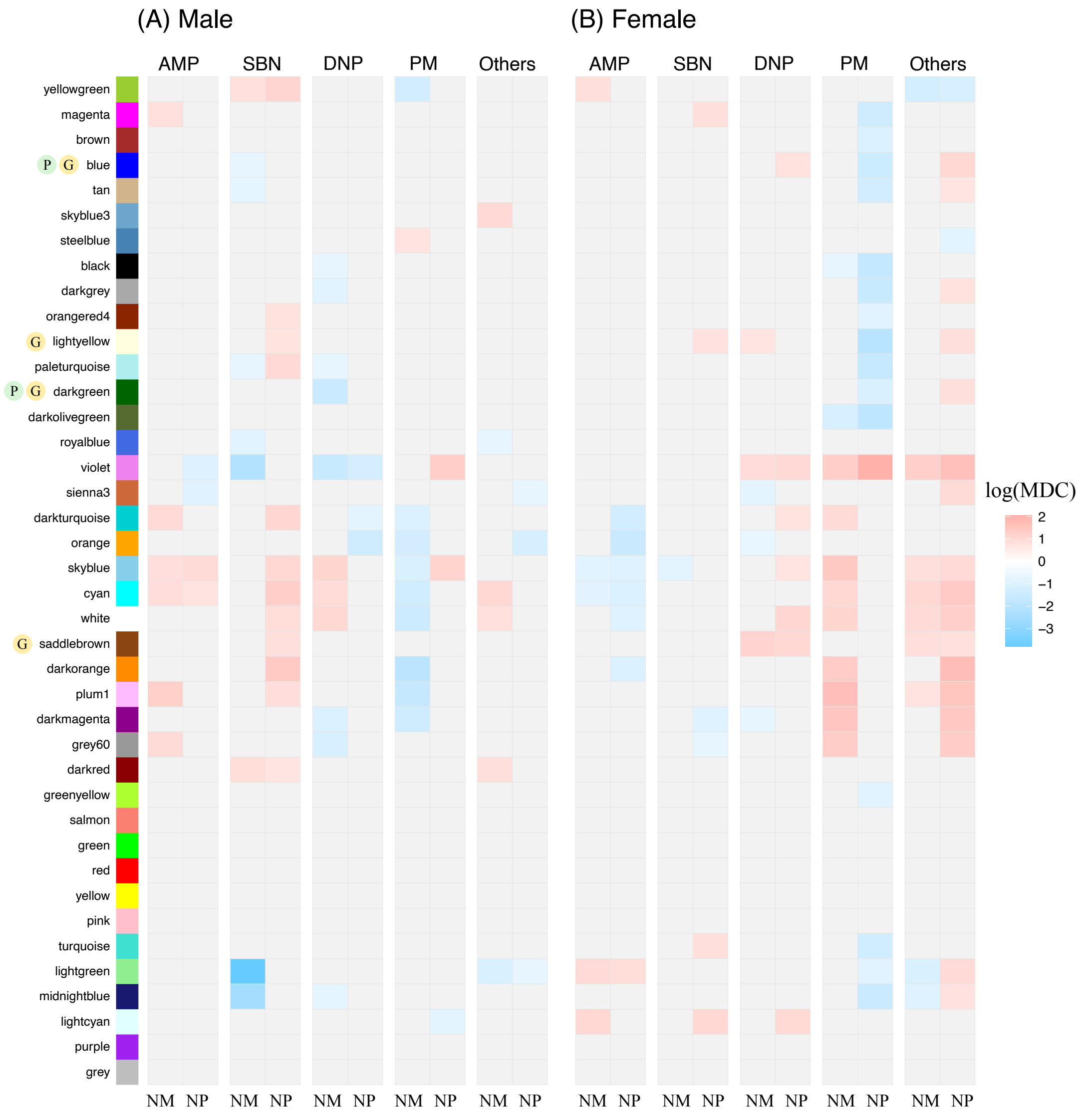

Figure S5. Co-expression gene modules with significantly different modular differential connectivity between treatments (MDC modules). Red cells indicate the modules showing significant gain of MDC in the E group compared against the NM or NP groups, and blue cells indicate the module showed significant loss of MDC. The modules over-represented with neuron projection and neurogenesis functions are marked with Ⓟ and Ⓖ, respectively.

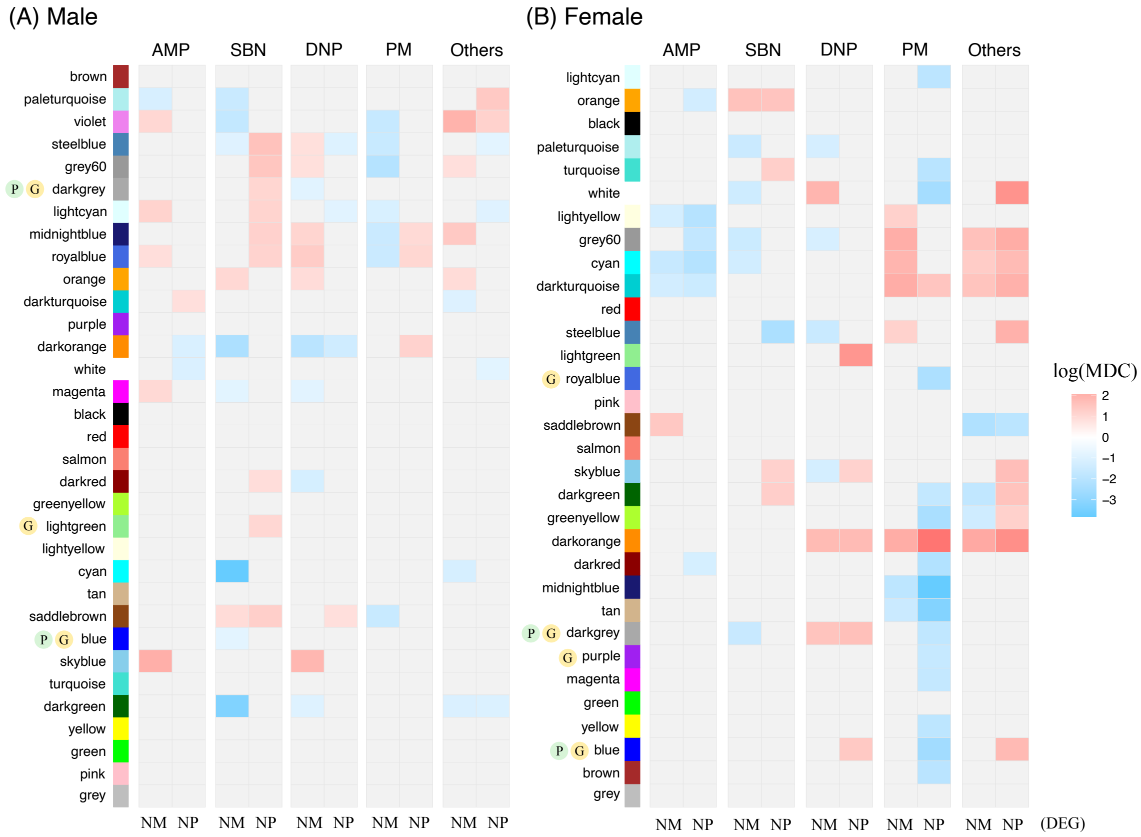

Figure S6. Gender-specific co-expression gene modules with significantly different modular differential connectivity between treatments (MDC modules). The co-expression modules are constructed with gender separated. Red cells indicate the modules showing significant gain of MDC in the E group compared against the NM or NP groups, and blue cells indicate the module showed significant loss of MDC. The modules over-represented with neuron projection and neurogenesis functions are marked with Ⓟ and Ⓖ, respectively.

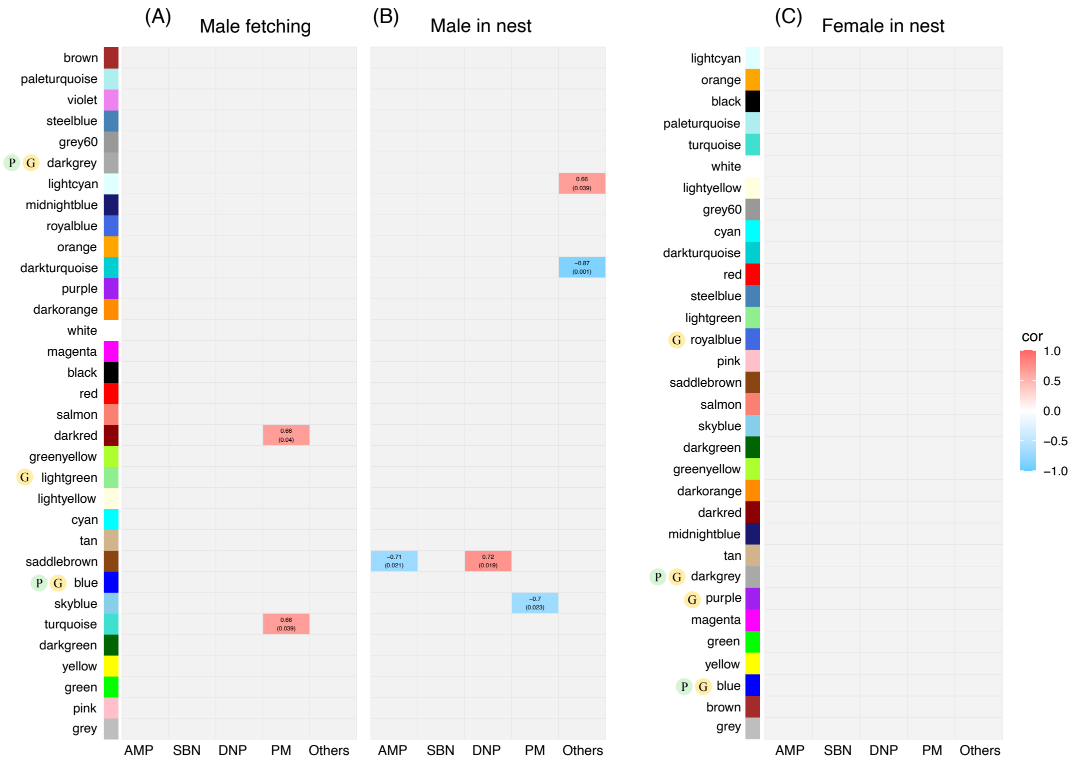

Figure S7. Gender-specific co-expression gene modules correlated to nesting behavior frequencies (NBF modules). The co-expression modules are constructed with gender separated. Colored cells indicate co-expression modules with significant correlations with the frequency of (A) fetching nest materials in males, (B) staying in the nest-box in males, or (C) staying in the nest-box in females in each brain region. The red color of the boxes indicates significantly positive correlation modules, and the blue color of the boxes indicates significant negatively correlation modules. Numbers within each cell indicate the correlation coefficient and the p-values (within brackets) of correlation analysis. The modules over-represented with neuron projection and neurogenesis functions are marked with Ⓟ and Ⓖ, respectively.

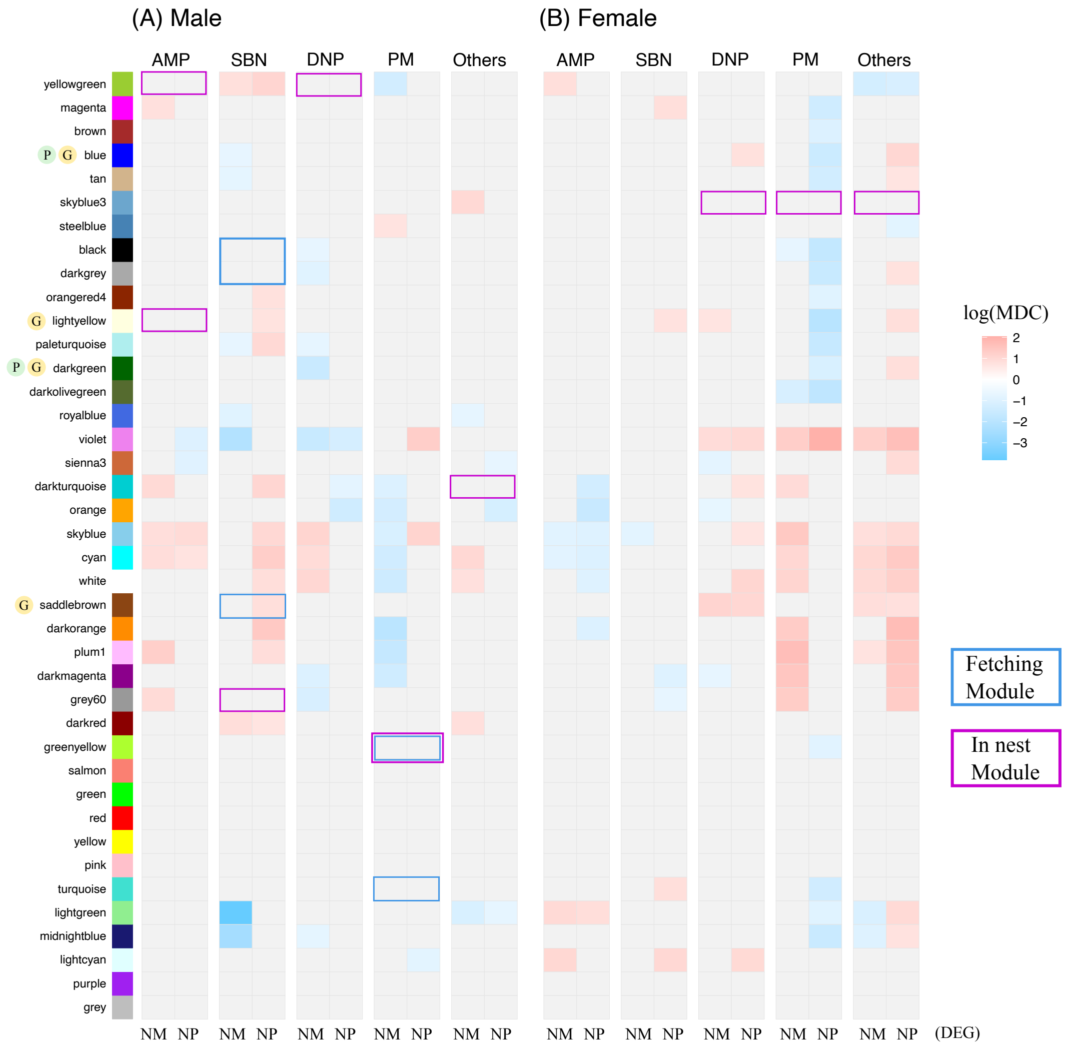

Figure S8. Comparison between molecular differential connectivity (MDC) modules and nesting behavior associated (NBF) modules. Solid red cells indicate the modules showing significant gain of MDC in the E group compared against the NM or NP groups, and solid blue cells indicate the module showed significant loss of MDC. Red empty boxes indicate co-expression modules with significant correlations with staying in the frequency of staying nest-box in males or females, and blue empty boxes for the frequency of fetching nest materials in males. The modules over-represented with neuron projection and neurogenesis functions are marked with Ⓟ and Ⓖ, respectively.

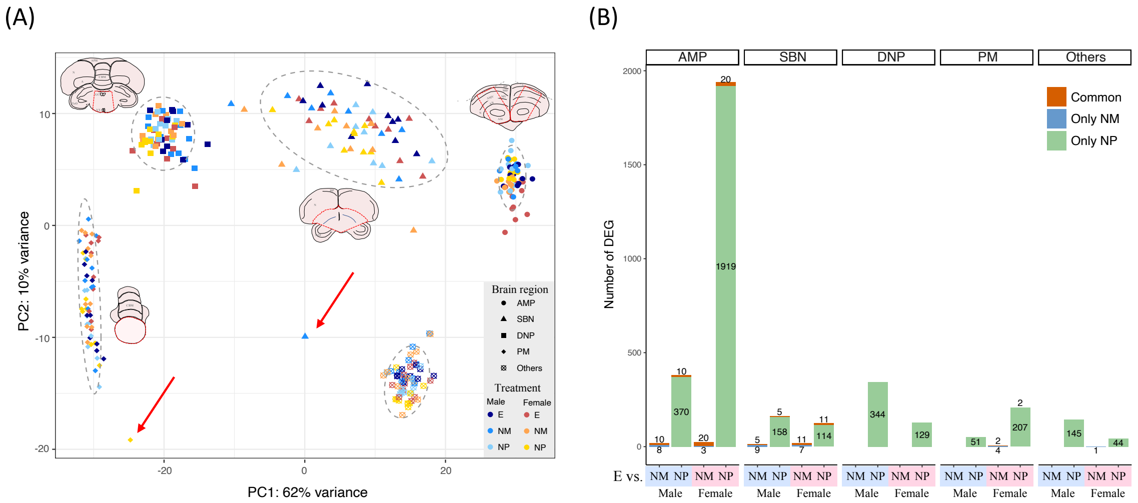

Figure S9. Brain transcriptomic clusters and DEGs with outlier removed. (A) Principal Component Analysis clusters of transcriptomes from five brain regions. Different colors indicate three treatments (E, NM and NP) and two genders, and different shapes indicate the five brain regions (AMP, SBN, DNP, PM and Others). Brain section images are positioned near to the transcriptomic clusters of their corresponding brain regions (see Fig. S2 for the detailed descriptions of the images). Red arrows indicate two seeming outliers, one from SBN and one from PM, which are moved from the dataset used for DEGs analysis. (B) The numbers of DEGs resulted from the dataset with the two outliers removed and the comparisons of E vs. NM and E vs. NP for each gender and brain region. DEGs that are exclusive to either the E vs. NM or E vs. NP comparison are represented in blue or green, respectively, and DEGs appeared in both the E vs. NM and E vs. NP comparisons are represented in red.
