# Supplementary material for: Brain Gene Regulatory Networks Coordinate Nest Construction in Birds
Source: Mol Biol Evol. 2024 Jun 25;41(7):msae125. doi: 10.1093/molbev/msae125 (PMC11223658; doi:10.1093/molbev/msae125)
Supplement: msae125_Supplementary_Data [file msae125_supplementary_data.zip › Supplementary Results_MBE_Final.pdf]

Brain gene regulatory networks coordinate nest construction in birds

Yi-Ting Fang[1,2], Hao-Chih Kuo[1]†, Cheng-Yu Chen[1,3]†, Shen-Ju Chou[4]†, Chia-Wei Lu[1] and Chih-Ming Hung[1]*
[1] Biodiversity Research Center, Academia Sinica, Taipei, Taiwan
[2] Department of Life Science, National Taiwan Normal University, Taipei, Taiwan
[3] Department of Life Science, National Taiwan University, Taipei, Taiwan
[4] Institute of Cellular and Organismic Biology, Academia Sinica, Taipei, Taiwan

* Corresponding author: Chih-Ming Hung
Email: cmhung@gate.sinica.edu.tw

Supplementary Results

Relationship between nest construction actions and nest materials used

We conducted the Pearson correlation analysis to investigate the potential correlation between the weight of nest materials used during nest construction and the frequencies of nesting actions for birds from the E group (see Methods). We found a significantly positive correlation between the frequency of male fetching actions and the weight of nest materials used during nest construction in the whole experiment trials ($p = 0.042$). In addition, there was a marginally positive correlation between the frequency of male fetching actions and materials used on the sacrifice day ($p = 0.086$; Fig. S1). In contrast, no significant correlation was observed between the usage of nest materials and the frequency of staying in the nest-box for both genders ($p > 0.5$; Fig. S1). These results were expected because the more frequently male birds fetched, the more nest materials used. However, there might be variation in the amount of nest materials collected per fetching action among individuals, rendering the correlation somewhat marginal. In contrast, the action of staying in the nest-box did not directly contribute to nest material collection,

resulting in no correlation between the two variables in either males or females.

Brain transcriptome profiles corresponded to brain regions

We collected five different brain regions of each bird, including AMP, SBN, DNP, pons and medulla (PM), and the rest parts of brain tissues ("Others"), leading to a total of 300 samples for RNA-seq analysis. The average number of raw read pairs per sample was 42 million (ranging from 31 to 60 million) and the average number of read pairs per sample after trimming was 40 million (ranging from 30 to 58 million). The average mapping rate by Hisat2 was 92% (ranging from 89% to 93%; Table S3). The principal component analysis (PCA) of whole transcriptome data (for all 300 samples with all 22,150 genes) clearly separated the five brain regions with PC1 explaining 62% of the variance and PC2 explaining 10% (Fig. 2a).

Female-skewed gene expression associated with nest construction

We found the number of DEGs in female birds around 2.5 time more than that in male birds for both E-NM and E-NP comparisons (136 DEGs for female and 57 DEGs for male in E-NM comparison, and 3,045 DEGs for female and 1,143 DEGs for males in E-NP comparison). We found female-skewed patterns in all brain regions and treatment comparisons except that males showed more DEGs than females in DNP and "Others" brain regions in the E-NP comparison (373 male DEGs and 220 female DEGs in DNP, and 219 male DEGs and 74 female DEGs in Others; Fig. 2b).

Stronger nesting-associated gene expression response in the anterior mother pathway than other brain regions

For the DEGs identified in the comparison between E and NM male finches, we found 31 genes

in AMP, 26 in SBN, none in DNP, 1 in PM and none in Others; in the comparison between E and NM female finches, we identified 77 DEGs in AMP, 61 in SBN, 10 in DNP, 11 in PM and 9 in Others. For the DEGs identified in the comparison between E and NP male finches, we found 593 genes in AMP, 223 in SBN, 373 in DNP, 66 in PM and 219 in Others; in the comparison between E and NP female finches, we identified 2434 DEGs in AMP, 266 in SBN, 220 in DNP, 751 in PM and 74 in Others (Fig. 2b, Table S4).

Among male E-NP-specific DEGs, 577 genes could be found in AMP, 213 in SBN, 373 in DNP, 66 in PM and 219 in Others; for female finches, we found 2374 E-NP-specific DEGs in AMP, 234, in SBN, 216 in DNP, 746 in PM and 72 in Others. Among the E-NM-specific DEGs in male finches, 15 genes could found in AMP, 16 in SBN, none in DNP, one in PM and none in Others; for female finches, we found 17 E-NM-specific DEGs in AMP, 29 in SBN, 6 in DNP, 6 in PM and 7 in Others. Among the common DEGs in male birds, 16 were identified in the AMP, 10 in SBN, and none in DNP, PM, or Others; in female birds, we identified 60 common DEGs in AMP, 32 in SBN, 4 in DNP, 5 in PM, and 2 in Others (Fig. 2b, Table S4).

Neurotranscriptomes revealed little signal of in situ neurogenesis in bird nest construction

To further examine the origin of added neurons during nesting, we subdivided the "neurogenesis" GO function group into "*in situ* neurogenesis" and "other neurogenesis actions" (Methods). Notably, our over-representation analysis showed no significant over-representation of *in situ* neurogenesis function in DEGs across any examined brain regions in male or female birds ($p > 0.05$, Fig. S4, Table S6).

Gene co-expression modules associated with the onset of nesting stage

We identified WGCNA modules that showed differential strength of expression connectivity

between nesting (i.e., E) and non-nesting (i.e., NM or NP) birds based on estimates of modular differential connectivity (MDC; Zhang et al. 2013). If a module showed significant MDC, we assumed it functionally relevant to shift in nesting condition and denoted it as a MDC module. In the E-NM comparison of male birds, six, nine, 11 and 10 out of the 39 WGCNA modules showed significant MDC in AMP, SBN, DNP and PM brain regions, respectively; in the male E-NP comparison, we found four, 12, three and three MDC modules in AMP, SBN, DNP and PM regions, respectively (Fig. S5, Table S10). In the female E-NM comparison, we identified five, one, six and 11 MDC modules in the AMP, SBN, DNP and PM regions, respectively; in the female E-NP comparison, seven, six, seven and 16 MDC module in the AMP, SBN, DNP and PM regions, respectively (Fig. S5, Table S10).

WGCNA modules associated with the frequencies of nesting behaviors

To identify the WGCNA modules that determined the frequencies of nesting behaviors, we estimated module eigengenes (MEs) based on individual modules' first principal components (Table S11). We then correlated MEs to the frequencies of fetching nest materials and staying in the nest-box in male E birds and the frequencies of staying in the nest-box in female E birds for each brain region. We denoted modules that showed significant correlations with the nesting behavior frequencies as "NBF modules".

Among the 40 modules, the fetching frequencies of male birds showed significant correlations with three modules in the SBN region and two modules in the PM region ($p < 0.05$; Fig. 4a). For male birds, the frequencies of staying-in-nest showed significantly negative correlations with two modules in AMP, one in SBN, and one in PM, alongside significantly positive correlations with one module in DNP (and one in Others) ($p < 0.05$; Fig. 4b). For female birds, the frequencies of staying-in-nest showed a significantly negative correlation with the

same module in DNP, PM (and Others) regions ($p < 0.05$; Fig. 4c).

MDC and NBF modules identified from gender-specific WGCNA modules

We conducted separate WGCNA analyses on male and female data, resulting in the construction of 33 WGCNA modules for males and 32 modules for females. Among the 33 male modules, two were enriched in both neuron projection-related and neurogenesis-related genes, while one module was only enriched in neurogenesis-related genes (Fig. S6a). In the 32 female modules, two were enriched in both neuron projection-related and neurogenesis-related genes, while two modules were solely enriched in neurogenesis-related genes (Fig. S6b). From the WGNCA modules, we then identified those showing either differential expression connectivity between groups that varied in the nesting status (MDC modules) or correlations with nesting actions (NBF modules). We found the general patterns of the new analyses with males and females separated were similar to the original ones with both sexes combined. However, inter-group comparisons were more complex because we needed to consider the different modules between male and female birds.

Among the gender-separated WGCNA that were associated with neurogenesis and neuron projection, female birds had relatively more MDC modules across the examined brain regions (excluding “Others”; module x brain region = 8 out of 32 for neurogenesis and 6 out of 16 for neuron projection) than did male ones (4 out of 24 for neurogenesis and 3 out of 16 for neuron projection; Fig. S6). The E-NP comparison showed relatively more MDC modules associated with neurogenesis (module x brain region = 6 out of 16 in females and 2 out of 12 in males) than the E-NM comparison (2 out of 16 in females and 2 out of 12 in males). We also found relatively more MDC module associated with neuron projection in the E-NP comparison (module x brain region = 4 out of 8 in females and 1 out of 8 in males) than E-NM comparison (2 out of 8 in

females and 2 out of 8 in males) although the difference was milder. In addition, female MDC modules were enriched with neurogenesis/neuron projection in more brain regions (i.e., SBN, DNP and PM) than male ones (i.e., SBN, DNP), the same pattern as those of analyses with both sexes combined.

In the gender-specific WGCNA modules, we also identified more NBF modules associated with male staying-in-nest action (module x brain region = 3 out of 132) than those associated with female staying-in-nest action (0 out of 128) or male fetching action (2 out of 132; Fig. S7). NBF modules for male staying-in-nest action were also detected in more brain regions (AMP, DNP and PM, excluding Others) than those for female staying-in-nest action (none) or male fetching action (PM).

Considering the similar patterns observed when separating gender compared to analyzing all 300 samples together, we believe that conducting WGCNA with all 300 samples offers more advantages and can provide more information than separating by gender. First, by identifying modules that encompass both sexes, we can compare modules associated with nesting behavior between sexes straightforwardly. Second, including more samples for constructing the WGCNA modules should lead to a more accurate estimation of correlations during network construction. Therefore, we believe that conducting WGCNA comprehensively with all 300 samples is the most advantageous approach in our condition.

Social hormone DEGs were female-skewed and strongly mate-induced, while social hormone NBF modules were male-skewed

We found several female E-NP-specific DEGs associated with social hormone (oxytocin, vasopressin and dopamine) signaling, known to influence nesting behaviors in birds and mice (Klatt and Goodson 2013; Hall et al. 2015; Bendesky et al. 2017). For example, *OXT*, encoding

an oxytocin precursor, was an E-NP-specific DEGs in DNP. *AVP*, encoding arginine vasopressin, was another E-NP-specific DEG in AMP. *AVPR1A* and *AVPR2* (with two Ensembl IDs, Table S4), encoding vasopressin receptors, were identified in PM and AMP, SBN and DNP, respectively. Additionally, three female E-NP-specific DEGs—*DRD1*, *DRD2* and *DRD5*—in AMP encoded for dopamine receptors; although *DRD2* and *DRD5* were also male E-NP specific DEGs, they were detected in males' Others region (Table S4). The results suggest the involvement of oxytocinergic, vasopressinergic and dopaminergic systems, induced by mates, in preparing female zebra finches for nesting.

One NBF (turquoise) module for male fetching behavior identified in PM contained *AVPR1A*, *DRD3* (another dopamine receptor gene) and *DBH*, which regulates dopamine to norepinephrine conversion (Kim et al. 2002; Table S8). In AMP, one NBF (lightyellow) module for male staying-in-nest behavior contained *TH* (with two Ensembl IDs, Table S8) involved in dopamine synthesis (Daubner et al. 2011), and it was enriched in functions related to dopaminergic synaptic transmission and mating behavior (Table S12). The results suggest that the vasopressinergic and dopaminergic systems may regulate male nest construction actions in the nesting stage, while not influencing female finches.

DEGs, MDC and NBF modules associated with tactile sensorimotor signaling in PM and beyond

We identified E-NP-specific DEGs related to the tactile sense, such as *DRGX* and *TLX3* in the female PM region (Table S4). *DRGX* (= *DRG11*), regulated by *TLX3*, is a transcription factor that patterns whisker-specific neuronal structure—barrelettes—in the principal trigeminal nucleus (PrV) of mice (Erzurumlu et al. 2010).

We also identified tactile-related, E-NP-specific DEGs in brain regions other than PM. For

example, in male AMP, we identified *PIEZO2*, which encodes a mechanosensitive ion channel and is critical to process tactile information received from duck beaks (Schneider et al. 2017) and *NTRK2*, which is critical to mechanoreceptor development (Calvavia et al. 2010) and shows strongly correlated expression with *PIEZO2* in the trigeminal ganglia of several duck species (Schneider et al. 2019). *KCNQ4* (with two Ensembl IDs, Table S4) in male DNP and SBN regions, encodes a potassium channel that modulates touch sensitivity in mice and humans (Heidenreich et al. 2012). In the male DNP region, *POU4F2* is an enriched transcription factor in mice' Merkel cells responding to light touch sensation (Haeberle et al. 2004), and also regulates the differentiation of neurons responding to gentle body touch in *Caenorhabditis elegans* (Duggan et al. 1998). *LHFPL5* in male AMP and DNP and *PCDH15* in both male and female AMP both encode mechanosensitive ion channels (Kefauver et al. 2020). *ASIC2* (with two Ensembl IDs, Table S4) in the female AMP region encodes a mechanosensitive ion channel in the rictus (beak base) of the common pigeon (*Columba livia*; Cabo et al. 2013).

Interestingly, many of the above DEGs were also included in BNF or MDC modules identified in PM. A fetching-associated BNF (turquoise) module in male PM included *PIEZO2* and *ASIC2* (Fig. 4, Table S8). *ASIC2* (with a different Ensembl ID than the one in the turquoise module)*, NTRK2* and *PCDH15* were included in one (blue) module and *LHFPL5* in another (brown) module, both of which were MDC modules in female PM (although the blue module was also an MDC module identified in male SBN and female DNP and Others; Fig. S5, Table S8).

Hub genes of MDC and NBF modules largely associated with gene transcription

To find the main drivers of the MDC and NBF modules, we identified their hub genes (see Methods for details). For MDC modules, we found six hub genes (out of three modules) for

males and eight hub genes (out of three modules) for females (excluding modules identified in the Others region; Table S14). The hub genes of a male MDC modules in the AMP region are largely associated with the function of gene transcription. A hub gene, *SP1*, is an important transcription factor that regulates the transcription of several house-keeping genes (Samson and Wong 2002). Another hub gene, *HCFC1*, is regulated by several transcription factors, including *SP1* (Zoppè et al. 1996). A third hub gene of this module, *RBM14* (= *CoAA*), encodes a coactivator protein that regulates both promoter transcription activity and RNA splicing decisions (Auboeuf et al. 2004).

For NBF modules, we found nine hub genes (out of three modules) for male fetching behavior and five hub genes (out of two modules) for males to stay in the nest-box (excluding modules identified in the Others region; Table S13). Five hub genes in two NBF modules in SBN and one hub gene in another NBF module in PM for male fetching behavior also have functions related to transcription regulation. That is, in one module of SBN, *KMT2C* (= *MLL3*) encodes a coactivator for RNA synthesis (Dorighi et al. 2017), *RBM39* (= *CAPER*) regulates alternative splicing and activates the transcription of estrogen receptors (Mai et al. 2016; Jung et al. 2002), *MAML1* encodes a transcriptional coactivator for NOTCH signaling (Wu et al. 2000), and *NSD1* also acts as a transcriptional coactivator to regulate RNA polymerase II function (Lucio-Eterovic et al. 2010). In the second module in SBN, *RBWD3* involves the acetylation of histones and the initiation of transcription (Wang et al. 2021). *LPIN1*—a hub gene of another module (the turquoise module) in the PM region—encodes a transcriptional coactivator (Péterfy et al. 2010) and is also found to affect muscle and motor neuron development (Lu et al. 2021). The above results indicate that genes involved in transcriptional regulation may play a role in altering the gene co-expression network connectivity between nesting and non-nesting conditions and driving gene co-expression networks that regulate fetching behavior.

In addition, a hub gene of one NBF model (the turquoise module) for male fetching behavior in the PM region, *SLC6A11*, encodes the GABA-transporter 3 (GAT-3) and may regulate extracellular GABA concentration, causing changes in GABAergic tonic conductance (Kersanté et al. 2013). GABA is one of the fundamental neurotransmitters in the central nervous system, regulates post-synaptic potential (Krnjević and Schwartz 1966) and is associated with the modification of neural firing patterns, network activity, and synaptic plasticity in the brain (Foster and Kemp 2006). Studies have found that dysregulating GAT-3 may cause changes in behavior, such as abnormal self-grooming or depression-like behavior in rats or mice (Zink et al. 2009; Yu et al. 2018). Our results suggest that GAT-3 may also play a role in regulating the fetching behavior of male nesting birds.

Supplementary References

Auboeuf D, Dowhan DH, Li X, Larkin K, Ko L, Berget SM et al. 2004. CoAA, a nuclear receptor coactivator protein at the interface of transcriptional coactivation and RNA splicing. *Mol Cel Biol* **24:** 442–453.

Bendesky A, Kwon Y-M, Lassance J-M, Lewarch CL, Yao S, Peterson BK, He MX, Dulac C, Hoekstra HE. 2017. The genetic basis of parental care evolution in monogamous mice. *Nature* **544**: 434–439.

Cabo R, Gálvez A, Laurà R, San José I, Pastor JF, López-Muñiz A et al. 2013. Immunohistochemical detection of the putative mechanoproteins ASIC2 and TRPV4 in avian herbst sensory corpuscles. *Anat Rec* **296:** 117–122.

Calavia MG, Feito J, López-Iglesias L, de Carlos F, García-Suarez O, Pérez-Piñera P et al. 2010. The lamellar cells in human Meissner corpuscles express TrkB. *Neurosci Lett* **468:** 106–109.

Daubner SC, Le T, Wang S. 2011. Tyrosine hydroxylase and regulation of dopamine synthesis. *Arch Biochem* **508:** 1–12.

Dorighi KM, Swigut T, Henriques T, Bhanu NV, Scruggs BS, Nady N et al. 2017. Mll3 and Mll4 Facilitate Enhancer RNA Synthesis and Transcription from Promoters Independently of H3K4 Monomethylation. *Mol Cell* **66:** 568–576.e4.

Duggan A, Ma C, Chalfie M. 1998. Regulation of touch receptor differentiation by the

Caenorhabditis elegans mec-3 and unc-86 genes. *Dev* **125:** 4107–4119.

Erzurumlu RS, Murakami Y, Rijli FM. 2010. Mapping the face in the somatosensory brainstem. *Nat Rev Neurosci* **11:** 252–263.

Foster AC, Kemp J. 2006. Glutamate- and GABA-based CNS therapeutics. *Curr Opin Pharmacol* **6:** 7–17.

Haeberle H, Fujiwara M, Chuang J, Medina MM, Panditrao MV, Bechstedt S et al. 2004. Molecular profiling reveals synaptic release machinery in Merkel cells. *Proc Natl Acad Sci* **101:** 14503–14508.

Hall ZJ, Healy SD, Meddle SL. 2015. A Role for Nonapeptides and Dopamine in Nest-Building Behaviour. *J Neuroendocrinol* **27**: 158–165.

Heidenreich M, Lechner SG, Vardanyan V, Wetzel C, Cremers CW, De Leenheer EM et al. 2012. KCNQ4 K+ channels tune mechanoreceptors for normal touch sensation in mouse and man. *Nat Neurosci* **15:** 138–145.

Jung D-J, Na S-Y, Na DS, Lee JW. 2002. Molecular Cloning and Characterization of CAPER, a Novel Coactivator of Activating Protein-1 and Estrogen Receptors. *J Biol Chem* **277:** 1229–1234.

Kefauver JM, Ward AB, Patapoutian A. 2020. Discoveries in structure and physiology of mechanically activated ion channels. *Nature* **587:** 567–576.

Kersanté F, Rowley SCS, Pavlov I, Gutièrrez-Mecinas M, Semyanov A, Reul JMHM et al. 2013. A functional role for both γ-aminobutyric acid (GABA) transporter-1 and GABA transporter-3 in the modulation of extracellular GABA and GABAergic tonic conductances in the rat hippocampus. *J Physiol* **591:** 2429–2441.

Kim CH, Zabetian CP, Cubells JF, Cho S, Biaggioni I, Cohen BM et al. 2002. Mutations in the dopamine β-hydroxylase gene are associated with human norepinephrine deficiency. *Am J Med Genet* **108:**140–7.

Klatt JD, Goodson JL. 2013. Sex-specific activity and function of hypothalamic nonapeptide neurons during nest-building in zebra finches. *Horm Behav* **64**: 818–824.

Krnjević K, Schwartz DS. 1966. Is γ-Aminobutyric Acid an Inhibitory Transmitter. *Nature* **211:** 1372–1374.

Lu S, Lyu Z, Wang Z, Kou Y, Liu C, Li S, Hu M, Zhu H, Wang W, Zhang C, et al. 2021. Lipin 1 deficiency causes adult-onset myasthenia with motor neuron dysfunction in humans and neuromuscular junction defects in zebrafish. *Theranostics* **11:** 2788–2805.

Lucio-Eterovic AK, Singh MM, Gardner JE, Veerappan CS, Rice JC, Carpenter PB. 2010. Role for the nuclear receptor-binding SET domain protein 1 (NSD1) methyltransferase in coordinating lysine 36 methylation at histone 3 with RNA polymerase II function. *Proc Natl Acad Sci* **107:** 16952–16957.

Mai S, Qu X, Li P, Ma Q, Cao C, Liu X. 2016. Global regulation of alternative RNA splicing by the SR-rich protein RBM39. *Biochim Biophys Acta* **1859:** 1014–1024.

Péterfy M, Harris TE, Fujita N, Reue K. 2010. Insulin-stimulated Interaction with 14-3-3 Promotes Cytoplasmic Localization of Lipin-1 in Adipocytes. *J Biol Chem* **285:** 3857–3864.

Samson SL, Wong NC. 2002. Role of Sp1 in insulin regulation of gene expression. *J Mol Endocrinol* **29:** 265–279.

Schneider ER, Anderson EO, Feketa VV, Mastrotto M, Nikolaev YA, Gracheva EO et al. 2019. A cross-species analysis reveals a general role for Piezo2 in mechanosensory specialization of trigeminal ganglia from tactile specialist birds. *Cell Rep* **26:** 1979–1987.

Schneider ER, Anderson EO, Mastrotto M, Matson JD, Schulz VP, Gallagher PG et al. 2017. Molecular basis of tactile specialization in the duck bill. *Proc Natl Acad Sci* **114:**13036–13041.

Wang X, Wang H-Y, Hu G-S, Tang W-S, Weng L, Zhang Y et al. 2021. DDB1 binds histone reader BRWD3 to activate the transcriptional cascade in adipogenesis and promote onset of obesity. *Cell Rep* **35:** 109281.

Wu L, Aster JC, Blacklow SC, Lake R, Artavanis-Tsakonas S, Griffin JD. 2000. MAML1, a human homologue of Drosophila Mastermind, is a transcriptional co-activator for NOTCH receptors. *Nat Genet* **26:** 484–489.

Yu X, Taylor AMW, Nagai J, Golshani P, Evans CJ, Coppola G et al. 2018. Reducing Astrocyte Calcium Signaling In Vivo Alters Striatal Microcircuits and Causes Repetitive Behavior. *Neuron* **99:** 1170–1187.e9.

Zhang B, Gaiteri C, Bodea LG, Wang Z, McElwee J, Podtelezhnikov AA et al. 2013. Integrated systems approach identifies genetic nodes and networks in late-onset Alzheimer's disease. *Cell* **153:** 707–720.

Zink M, Vollmayr B, Gebicke-Haerter PJ, Henn FA. 2009. Reduced Expression of GABA Transporter GAT3 in Helpless Rats, an Animal Model of Depression. *Neurochem Res* **34:** 1584–1593.

Zoppè M, Frattini A, Faranda S, Vezzoni P. 1996. The complete sequence of the host cell factor 1 (HCFC1) gene and its promoter: a role for YY1 transcription factor in the regulation of its expression. *Genomics* **34:** 85–91.
